# Conceptualisation of health among young people: a systematic review and thematic synthesis of qualitative studies

**DOI:** 10.1136/bmjph-2024-001648

**Published:** 2025-06-25

**Authors:** Katrin Metsis, Joanna Inchley, Andrew James Williams, Sebastian Vrahimis, Lamorna Brown, Frank Sullivan

**Affiliations:** 1 School of Medicine, University of St Andrews, St Andrews, UK; 2 School of Health and Wellbeing, University of Glasgow, Glasgow, UK; 3 School of Health in Social Science, University of Edinburgh, Edinburgh, UK

**Keywords:** Public Health, Preventive Medicine, Social Medicine

## Abstract

**ABSTRACT:**

**Introduction:**

Self-reported health is a widely used measure of general health in survey research. Qualitative studies that investigate young people’s conceptualisation of health are hard to locate and use different measures of health and sample construction. This review aims to synthesise the findings of qualitative studies that investigate how young people conceptualise their health, including during self-assessments.

**Methods:**

We searched MEDLINE (Ovid), PsycINFO (APA PsycNet), ProQuest Sociology Collection (Applied Social Sciences Index & Abstracts/Sociological Abstracts/Sociology Database) and Web of Science Core Collection without date restrictions. Searches were last updated on 11 March 2025. We searched the reference lists of relevant studies and conducted backward and forward citation searching. Papers reporting qualitative primary studies that focused on the conceptualisation of health among 10–24-year olds were included. We used the Quality Framework for quality appraisal and the thematic synthesis strategy for data synthesis.

**Results:**

Twenty-one studies from 11 countries with a pooled sample of 1434 participants met the inclusion criteria. We developed two analytical themes: (1) ‘dimensions of health’ and (2) ‘health in context’ with eight subthemes. Factors from the physical dimension of health, such as symptoms, physical activity or diet, dominate in young people’s conceptualisation of health; these factors are also considered when responding to self-assessed general health questions in the surveys. When the survey question uses the word ‘feel’, respondents discuss elements from physical, social and mental dimensions, and their interaction in the formation of health. In some studies, young people describe health in relation to context.

**Conclusions:**

This is the first systematic review of the conceptualisation of health among young people. Our findings indicate that self-reported general health questions in the surveys invite young people to focus on the physical aspects of health. Overall, young people hold a holistic conceptualisation of health. To improve the understanding of young people’s health, future research needs to focus on conceptual clarity. Different wording captures different aspects of health that need to be balanced for optimal development of young people’s health.

**PROSPERO registration number:**

CRD42022367519.

**WHAT IS ALREADY KNOWN ON THIS TOPIC**

- Self-reported general health is a widely used measure in survey research. Studies of the adult population show that the most frequent referents in self-ratings are from the physical dimension of health. For young people, similar studies are hard to locate and often apply inconsistent terminology.

**WHAT THIS STUDY ADDS**

- This is the first systematic review that focuses on the conceptualisation of health among 10–24-year olds. It facilitates the interpretation of the research that utilises self-rated general health questions.

**HOW THIS STUDY MIGHT AFFECT RESEARCH, PRACTICE OR POLICY**

- Different terms, such as ‘health’ or ‘feel’ in the self-rated general health questions measure different dimensions of health.
- We recommend that primary qualitative studies use reporting guidelines. This is needed for conceptual clarity, quality appraisal and application and synthesis of results.
- Good physical health requires an optimal social and contextual environment. This review draws attention to the health-harming impact of practices such as achievement groupings in schools or the persistent impact of adverse experiences.

## Introduction

 Self-reported health (SRH), also referred to as self-assessed or global health, is a widely used indicator in surveys and questionnaires. Its usefulness is based on its association with mortality and morbidity measures such as functional health, allostatic load and utilisation of health services.^1^,^5^ SRH is a useful indicator for studying health in young people. Clinical diagnoses are uncommon at this age; however, many diseases are initiated long before clinical symptoms, and many risk factors are present in adolescence.^6 7^ The WHO has estimated that up to 70% of premature deaths among adults are linked to behaviours initiated during adolescence.^8^

It is known that self-assessments arise from cognitive processes that summarise different aspects of health, such as symptoms or perceived risks of future health, and are embedded in social and cultural contexts.^3 9^ Therefore, the meaningful interpretation of secondary data analysis that utilises SRH measures requires a clear understanding of how respondents interpret their bodily information during self-assessments.^2 3^ Qualitative studies uncover health factors reported by respondents, while quantitative research tends to present respondents with a range of preselected factors.^2 3 9^ In adult studies, the most frequent referents in self-ratings are from the physical dimension, such as health problems or functional ability.^10^,^14^ People also consider health behaviours and social and mental aspects of health, such as fitness and relationships.^10^,^16^

The evidence on young people’s conceptualisation of health is patchy, and qualitative evidence is challenging to locate in database searches.^17^ It is often studied in the context of certain health behaviours such as diet, or by applying related concepts such as well-being.^18^,^33^ Often, different age groups are included.^34^,^42^ Similarly to adult studies, the most frequently mentioned factors are from the physical domain such as health behaviours, symptoms, diagnoses, lack of illness, functionality or physical appearance.^18^,^2124 25 27^ Respondents also view health as a holistic concept^2125^,^27 34 38 40 41^ and acknowledge the role of social relationships,^22^,^2534^ religion and traditions,^22^,^2435 36 41^ physical environment,^22 26 33^ violence^25^ and racism.^41^ Quantitative studies have identified factors from physical, mental and social dimensions of health, and health as a holistic concept.^43^,^45^

Therefore, this review was warranted for several reasons. An overarching aim was to systematically identify and synthesise qualitative studies that investigate the conceptualisation of ‘health’ among 10–24-year olds. This is needed for improved interpretation of survey findings, which use self-rated general health questions. Our focus is on the conceptualisation of ‘health’ as a distinct term; research has shown that related terms such as well-being, quality of life or health-related quality of life are conceptualised differently.^46^ Second, the review focuses on 10–24-year olds. This age definition of young people is used in official statistics, and we know that health ratings and considered factors vary by age.^10 39 47^ Third, focusing on young people’s health is overall important. Adolescence is a period of rapid physical and social maturation; optimal health is required to successfully navigate this transition.^648^,^50^ Although adolescence is a healthy life period compared with younger children and adults, neglecting young people’s health diminishes returns from investments in maternal and child health.^49^ Moreover, adolescents are future parents and thus will be shaping the health of their children and the next generations.^6 49^

This review aimed to identify and synthesise the findings of qualitative studies that investigate young people’s conceptualisation of health.

1. How do young people reason when they answer self-reported general health questions in the surveys?
2. How do young people reason when they rate health as very good, good, fair or bad?
3. How do young people understand the concept of health generally?

## Materials and methods

The systematic review protocol was registered in PROSPERO (registration number CRD42022367519) and published.^51^ As an epistemological position, this review adopts a position of critical realism (CR).^52^,^54^ CR combines realist ontology with relativist epistemology, which means that knowledge of reality is mediated by our perceptions and beliefs.^53^ Also, CR enables us to move away from the structure/agency dichotomy in understanding health-giving or harming practices.^52^,^54^ The review is reported following the Enhancing Transparency in Reporting the Synthesis of Qualitative Research checklist.^55^ (online supplemental file 1) The Preferred Reporting Items for Systematic Reviews and Meta-Analyses checklist is also included in online supplemental file 1.

### Search strategy and selection criteria

The review question, search strategy and inclusion criteria were framed in terms of the study population, intervention, comparator (not applicable here), outcomes and design.^56^ Table 1 summarises the inclusion and exclusion criteria.

**Table 1:** Inclusion and exclusion criteria

	Inclusion criteria	Exclusion criteria
Study population	Young people and adolescents with or without health conditions	Wider age range if results for 10–24 year-olds cannot be differentiated
Intervention	Conceptualisation of health in survey context or overallThe study question includes the term ‘health’ or ‘feel’	Related concepts of well-being, quality of life, health-related quality of life
Study outcome	‘Conceptualisation of health’: synthesis of factors discussed by young people when they discuss the concept of health	Not reporting outcomes of interestInsufficient detail for data synthesisConceptualisation of health in the context of certain phenomena such as physical activity or alongside concepts such as illness
Study design	Qualitative studiesVisual methods (photography, mind maps, drawings)Written descriptions of drawings or photographsWritten data from open-ended questions that were analysed by qualitative methods (eg, content analysis)Mixed methods studies if it is possible to distinguish qualitative findings	Quantitative studiesMixed method studies if it is not possible to distinguish between qualitative and quantitative findingsConference abstracts, opinion piecesBook reviewsStudies without any empirical aspect

Systematic searches were conducted on the MEDLINE (Ovid), PsycINFO (APA PsycNet), ProQuest Sociology Collection (Applied Social Sciences Index & Abstracts/Sociological Abstracts/Sociology Database) and Web of Science Core Collection. All search strategies are available in online supplemental file 2. We did not apply date restrictions. The search timeline was as follows: (1) preliminary search in Medline was conducted in January 2022; (2) preliminary searches in other databases were completed in May and June 2022; (3) all searches were updated in March 2023 and database search alerts were set up; (4) all searches were updated on 24 April 2024 and database search alerts were renewed. As of 11 March 2025, no additional studies have been identified. We applied an iterative search strategy and combined keywords and Medical Subject Headings (MeSH). The key search terms were ‘self-assessment’, ‘health status’ and ‘adolescent’. Key words and MeSH were combined using the ‘OR’ and ‘AND’ operators, and search strategies were complemented iteratively with the terms identified from retrieved studies. We applied the techniques of reference checking and conducted backward and forward citation searching through Google Scholar. Research has shown that these techniques complement database searches in social science systematic reviews and increase the number of identified studies.^57^

The study population was ‘young people’ aged 10–24 years; the term ‘adolescents’ refers to 10–19-year olds.^8^ We included qualitative studies where the primary aim was to investigate the conceptualisation of ‘health’. Studies investigating related concepts such as ‘well-being’, ‘quality of life’ or ‘health-related quality of life’ were excluded because research has shown that these are conceptualised differently compared with the SRH.^46 51^ Studies that investigated the conceptualisation of health in the context of certain health behaviours, such as healthy eating or physical activity, were excluded because they invite respondents to explore health from a predefined perspective. Studies that used written data were included if data were analysed using qualitative methods. Mixed methods studies were included if it was possible to separate the findings of the qualitative component.^4758^,^60^ For those studies, only key findings of the qualitative component were extracted.

### Searches and data extraction

One researcher (KM) searched the databases and uploaded the title and abstract of retrieved studies into the EndNote (V.20) library. The same researcher screened all titles and abstracts for inclusion. A second researcher (SV) screened a random sample of 20% of the abstracts to establish the agreement between reviewers. The reasons for conflict were resolved in discussion. The data extraction grid was piloted during preliminary searches.^51^ The following data were extracted by one researcher (KM): study reference, country, the aim of the study, sample characteristics, health question, data collection and analysis methods, and key findings. A second and third researcher (SV and LB) independently reviewed all extracted data. We used a shared EndNote library and MS Excel and Word documents to manage this process.

### Methodological quality of the included studies

We used a modified version of the Quality Framework (the QF) to appraise included studies (online supplemental file 3); we removed question 5 specific to evaluation studies (How clear is the basis of evaluative appraisal).^61^ We adopted the grading system from Dyer *et al*: A (no or few flaws), B (some flaws), C (significant flaws) and D (untrustworthy).^62^ The first author (KM) allocated each question a grade, and the second and third researchers (SV and LB) independently reviewed these results. Even though some studies were rated as having low methodological quality in some respects, none of the studies overall was untrustworthy or with significant flaws. They all contributed relevant knowledge and were included in the review. The selection of the appraisal tool is described in the study protocol.^51^

### Data synthesis

The conceptual framework for the data synthesis was informed by (1) definitions of health that include physical, mental, social and cultural dimensions of health and (2) social determinants of health approach.^63^ ^64^ We used the thematic synthesis strategy by Thomas and Harden.^54^ Thematic synthesis allows the inclusion of studies that include ‘rich’ (eg, studies adopting ethnographic methodology) or ‘thinner’ (eg, studies using visual or written data) descriptions of the data.^55^ It is informed by CR and was developed to facilitate the methodologically sound synthesis of qualitative studies.^53 54^ We followed three stages of data synthesis: (1) coding of the findings of primary studies, (2) organising the codes into descriptive themes and (3) developing analytical themes.^54^ The process of developing the themes was inductive. We used NVivo software (Release 1.5.1) throughout three stages, and MS Excel and Word documents to support data analysis.

### Patient and public involvement

Patients and/or the public were not involved in the design, or conduct or reporting or dissemination plans of this research.

## Results

**Figure 1:**
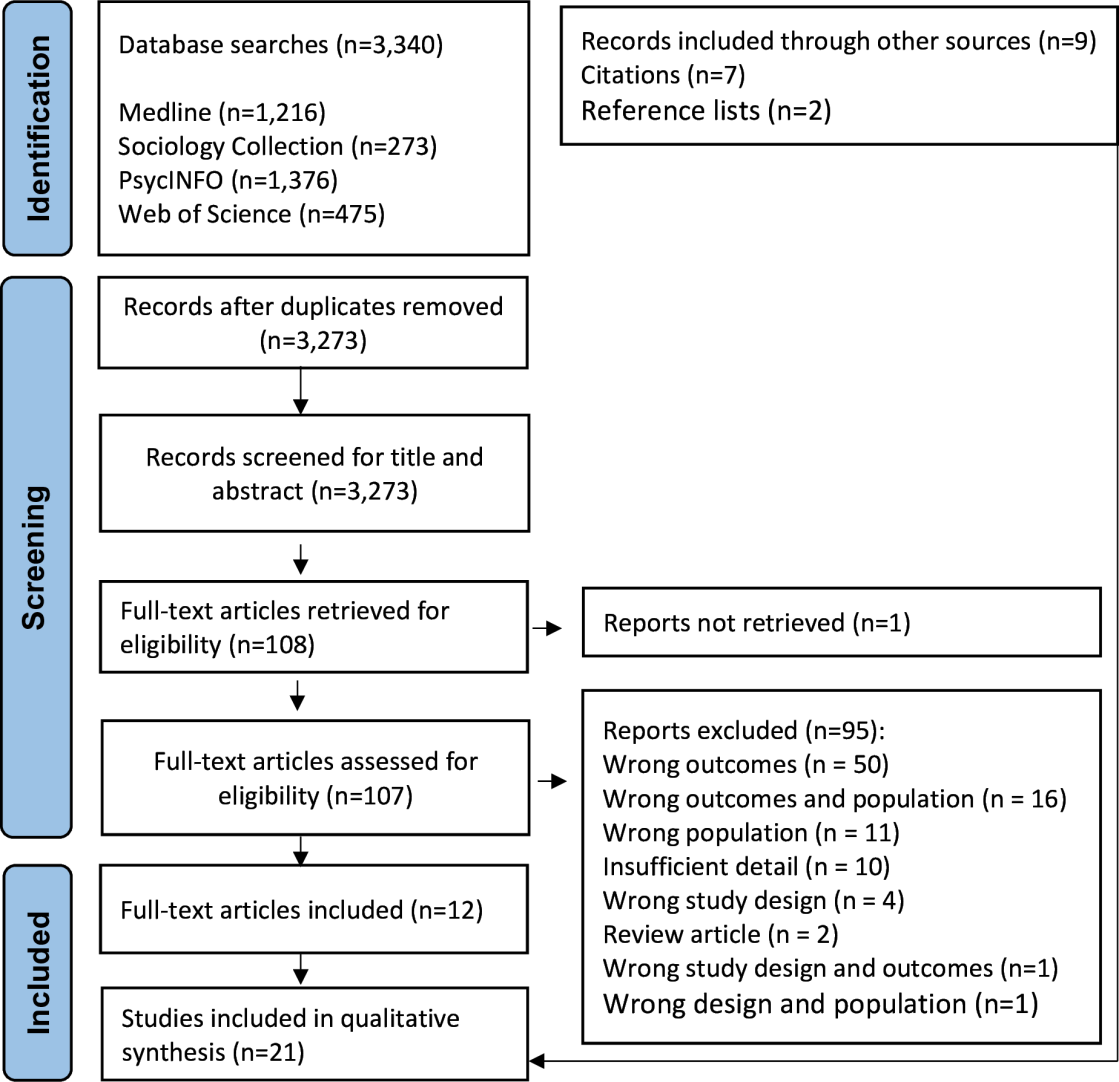
PRISMA flowchart of study identification and selection. PRISMA, Preferred Reporting Items for Systematic Reviews and Meta-Analyses.

The study screening and selection process is shown in figure 1. The database searches yielded 3340 studies. After duplicates were removed, 3273 studies were screened for the title and abstract. Of those, 108 studies were obtained for the full-text review. One study could not be located, and 95 studies were excluded after full-text review. It was not feasible to keep a count of the studies we retrieved from citation searching and reference lists; the final number of included studies from this method (n=9) is shown in figure 1. The decision to exclude some studies was complicated because health was studied alongside other phenomena, information was insufficient for synthesis or respondents’ age was close to the inclusion criteria. We felt that this process could be seen as arbitrary, therefore, all those studies are shown in online supplemental file 4.

Twenty-one studies were included in the synthesis. Twelve studies were located from the database searches,^4758 65^,^74^ two from the reference lists^75 76^ and seven from the citation searches.^5960 77^,^81^ Complete data extraction is shown in online supplemental file 5, and a description of the included studies is displayed in online supplemental file 6.

Included studies are summarised in table 2; they were categorised by the type of health question applied in the original studies:

1. Studies that investigated the concept of health in the survey context; the study question includes the word ‘health’.
2. Studies that investigated the concept of health in the survey context; the study question includes the word ‘feel’.
3. Studies that investigated the concept of health generally; the study question includes the word ‘health’.
4. Studies that investigated the concept of health generally; the study question includes the word ‘feel’.

**Table 2:** Description of included studies

Reference	Country	Aim	Sample
Studies that investigated the concept of health in the survey context; study question includes the word ‘health’ or ‘feel’			
Joffer *et al*^67^	Sweden	How adolescents interpret and reason when answering a question on self-rated health. 2) Contrasting ‘feel’ and ‘health’ in the SRH question.	Age: 12–13 and 17–18Male: N=29 Female: N=29
Välimaa^60^	Finland	How do 15-year-old adolescents understand the concept of health in the survey context?	Male: N=15 Female: N=12Age: 15 (9th grade pupils)
Studies that investigated the concept of health generally; study question includes the word ‘health’			
Berman^75^	Canada	How is health understood and experienced by two groups who have grown up amid violence, children of war and children of battered women?	Age 10–17Children of war: N=16Children of battered women N=16
Borraccino *et al*^65^	Italy	To explore the core categories evoked when adolescents describe what it means to be ‘healthy’ and ‘unhealthy’.	Age: mean 15.3Male: N=19 Female: N=15
Cetin *et al*^77^	Turkey	Determine the views of ninth-grade students about the concept of health.	9th-grade studentsMale N=81 Female N=75
Dow *et al*^78^	Ireland	How girls from disadvantaged communities make sense of “being healthy?”	Age: 10–12Girls only: N=22
Flick and Röhnsch^79^	Germany	Which representations of health do homeless adolescents hold?Which forms of health practices are reported or can be observed?	Age: 14–20Male N=12 Female N=12
Hager^80^	The US	What is the concept of health among adolescents with Insulin Dependent Diabetes Mellitus in a rural state?	Age: 12–18Male: N=7 Female: N=1
Hanna and Jacobs^66^	The US	What is the meaning of health among adolescents diagnosed with cancer?How is the meaning of health communicated with the use of photography?	Age: 14–17Male: N=3 Female: N=1
Hanna *et al*^81^	The US	To explore and describe the concept of health held by adolescents with diabetes.	Age: 12–19Male: N=6 Female: N=3
Hariharan *et al*^47^	India	How do school children conceptualise health?Does the concept of health show a progressive change across the age?	Age: 11–16N=667Male: 56% Female: 44%
Karabanow *et al*^58^	Canada	How homeless young people understand health and wellness, how they define good and bad health, and their experiences in accessing diverse health services.	Age 16–24Male: N=12 Female: N=3 N=10 health and social service providers
Michaelson *et al*^59^	Canada	What are adolescent perceptions of health?	Age: 12–15Male: N=13 Female: N=27
O’Higgins *et al*^69^	Ireland	What adolescents understand by words ‘health’ and ‘happy’.	Age: 13Male: N=16 Female: N=15
Ott *et al*^70^	The US	To describe adolescents’ ‘emic’ views of health and provide implications for state policy.	Age: 15–24Male: N=34 Female: N=34
Parvizy *et al*^76^	Islamic Republic of Iran	To gain an understanding of adolescents’ perspectives on health and develop a categorical model of health.	Age: 11–19Male: N=26 Female: N=26
Randell *et al*^71^	Sweden	How adolescent boys understand the concept of health and what they find important for its achievement.	Age: 16–17Boys only: N=33
Sasakamoose *et al*^72^	Canada	To explore the understanding of health among First Nations and Metis youth and the health of their communities.	Age: 14–17N=13
Woodgate and Leach^74^	Canada	How youth define health in the context of their life situations: how youth define health, what it means to be healthy, how life situations influence their health?	Age: 12–19Male: N=29 Female: N=42
Studies that investigated the concept of health generally; study question includes the word ‘feel’			
Larsson *et al*^68^	Sweden	Describe the phenomenon of health as experienced by adolescent girls in Sweden.	Age: 13–19Girls only: N=15
Spencer^73^	England	To elicit young people’s understanding of health and enquire how these meanings may be shaped within the context of everyday lives	Age 15–16Male: N=29 Female: N=26Professionals: N=18

SRH, self-reported health.

This categorisation was developed in line with the aims of this review: to facilitate the interpretation of survey findings and to improve the understanding of young people’s conceptualisation of health overall.

The quality appraisal was challenging because the required information was not always explicit, and we discussed this aspect a lot. This might have contributed to the rather high agreement in judgements (92%); this was calculated from the ratings of both reviewers to all QF questions. The results of the quality assessment are shown in online supplemental file 7. Studies that stand out by their quality ratings were in the format of peer-reviewed journal articles related to PhD theses,^67 73^ a PhD thesis manuscript^60^ and a Master’s thesis manuscript.^80^ All other studies were published as peer-reviewed articles and varied in terms of quality. The grade ‘significant flaws’ was mostly allocated to the following questions: (1) how well defended is the sample design/target selection of cases/documents (question 6); (2) contexts of data sources—how well they are retained and portrayed (question 10); (3) what evidence is there of attention to ethical issues (question 16) and (4) how adequately has the research process been documented (question 17). Questions 16 and 17 also attracted ‘untrustworthy’ ratings for two and one study, respectively. Despite this, none of the studies was untrustworthy overall, and all studies contributed new knowledge to the synthesis.

### Synthesis findings

- The three stages of thematic synthesis are visualised in online supplemental file 8. Manuscripts were entered into the NVivo project and grouped by four question types as discussed earlier. First, KM coded the Results sections of the primary studies line by line, resulting in 124 codes such as ‘sports’ or ‘family’. Second, KM compared and refined the codes across four questions; this resulted in 66 codes such as ‘diet’ or ‘school’. Third, KM combined similar codes into a hierarchical structure of descriptive themes (eg, ‘physical health’ or ‘emotions’). Coding was discussed with the study team. Fourth, the first set of analytical themes was developed by reviewing codes and descriptive themes and observing recurrent and contradictory ideas (phase 2–3 column in online supplemental file 8). Based on this, KM wrote the first draft of themes and discussed these with FS, AJW and JI.

The iterative process resulted in two analytical themes and eight subthemes:

1. Dimensions of health
  - Physical dimension: health behaviours, functionality and physical appearance.
    - Survey question: ‘How is your health in general?’
  - Social dimension: relationships with friends, family and (in) school.
  - Mental dimension: emotions and subjectivity of health.
  - Intersectionality of health and striving for balance.
    - Survey question: ‘How do you feel?’
2. Health in context
  - Physical context: Environment, housing, and safety.
  - Material resources.
  - Informational context and technology.
  - Cultural environment: traditions, culture and religion.

Online supplemental file 1 displays the representation of themes, subthemes and main factors in included studies. Box 1 shows the illustrative quotations for each theme.

Box 1 **Illustrative quotations**

**Dimensions of health**

Physical dimension: health behaviours, functionality and physical appearance.

- ‘I think my definition of health would be eating right, um keeping active, following the food guide and sleeping the right amount of time. You don’t want to oversleep or undersleep’.^74^
- ‘Sports and recreation influenced me to stay healthy, in shape and out of trouble.^72^
- ‘Quite often, I ask myself how it is, if you are really healthy. Or often I wish to be simply really fit, because everything is much easier then somehow’.^79^
- ‘We should brush our teeth two times a day, wash hands before eating and go to sleep timely’.^47^
- ‘I need to take my shots and test my blood to stay healthy’. (picture of insulin, syringe and glucometer)^80^

Survey question: ‘How is your health in general?’

- Boys focus group.^60^
- ‘So there is nothing permanent like compulsive movements or rheumatism …’
- ‘I rather thought about general fitness. And of course, what if you have a skin cancer …’ (Original quote in Finnish, see online supplemental file 10).
- … Health for me, it’s more about how I take care of my body and everything like that… purely chemical. Just how the body feels, but not how I feel as a person.^67^

Social dimension: relationships with friends, family and (in) school

- ‘A loving supportive family’ (picture of family around Christmas tree).^81^
- ‘Love and very good friends, and having people who believe in you, both in school, sports, and everything’.^71^
- ‘He [the teacher] helped me to find solutions to problems I never thought would be possible, making sense of it, his help made me become more self-confident. When getting this support you are pushed to continue working and to give your all’.^68^
- ‘Street kids are a bunch of messed up people…like with all the family stuff, like abuse and violence … that’s always with us, always on my mind’.^58^

Mental dimension: emotions and subjectivity of health

- ‘Being in love’ (picture of girlfriend).^66^
- ‘Well, my Dad’s a doctor, and he says that it’s important to enjoy yourself and have fun because that affects how healthy you feel’^69^
- ‘… it is that human is happy and gets along with other people …’.^77^
- Good health is not absolute: ‘You can be healthy without being completely healthy’.^59^

Intersectionality of health and striving for balance

- ‘But if you had a, like a healthy friend, like she could, or he could be like c'mon let us go to gym and, c'mon let us eat this salad and we can go into Chopped (salads take-away)…I'll see you tomorrow for your daily jog around the block’.^78^
- ‘There’s been instances that I didn’t feel very well. I felt really tired because emotionally I was exhausted. It’s just been overloading me. I haven’t been able to do anything. I didn’t go to school those days and I didn’t socialize with anybody. I sat at home and did nothing, and just sat there and thought about it [his father]. It’s really hampered me physically. I haven’t shown any signs of it but I’ve just been feeling really bad, and my stomach ache this morning I think was caused by emotional stress. …’.^75^
- ‘I wanted to do drama, but because I’m in this half I can’t do drama [y] people call us the dumb half and that’s why we call it the dumb half because we are, our confidence ain’t there any more [y] (focus group)’.^73^

Survey question: ‘How do you feel?’

- ‘I take it [homework] with me on the weekends… sometimes I even forget to eat… It’s too much, school takes up my life’.^67^
- ‘Many things influence health, mental balance, physical balance—everything. There are so many things, you cannot say what influences it and what doesn’t’.^60^ (Original quote in Finnish, see online supplemental file 10).

**Health in context**

Physical context: environment, housing and safety

- ‘Well it is kind of obvious, I mean a good community is like lots of trees, and people can run around and not get shot at and things like that, you know. There is greenery, and like people do not carry guns and shoot people all the time’.^74^
- ‘[W]e need to have aﬀordable housing that isn’t mouse infested and full of asbestos’.^58^

Material resources

- ‘(‘[…] he/she has his/her own bedroom’) and outside (‘[…] green space is available in which he/she can walk […] with friends’)’^65^
- ‘If you’re working however many jobs and school and everything, you don’t have time to make healthy foods … You throw a hot pocket in the microwave before you leave for work’.^70^

Informational context and technology

- ‘They say ‘Oh, you guys can’t buy sodas,’ but there are soda machines everywhere. Why would they have them if they don’t want us to buy them?’^70^

Cultural environment: traditions, culture and religion

- ‘Adolescents know that such shackles as religion, tradition, morality, and social norms could guarantee adolescents’ health relatively, but we want to try everything’.^76^
- ‘[Y]ou get harassed by every kind of group. You get harassed by suits for God’s sake. It’s not just teenagers, you know, it’s society’.^58^

### Theme 1: dimensions of health

Respondents differentiated between different types of health: physical, social and mental health:

‘Well, I think there are a lot of different kinds of health, like not one big health. I think it’s a mix of all different kinds. So there’s like physical health where you like run and keep your body healthy, and there’s emotional health where like you make sure you’re telling people what’s on your mind and you’re not keeping everything all bottled up and you’re not sad all the time and stuff. Then mental health, ’cause you like should have like a bit of smarts in your body’.^74^ (Girl)

#### Physical dimension: health behaviours, functionality and physical appearance

Factors from the physical dimension of health were identified in all studies. Physical activity or fitness were included in all studies except Parvizy *et al.*^76^ Only four studies did not mention diet.^68 72 73 76^ Ten studies referenced sleep^47 58 60 66 67 71 74 75 78 81^ and taking care of own body was identified as an element of health in seven studies.^4758 75 77 79^,^81^ Health as a functionality includes aspects such as the ability to achieve things or participate in activities; 12 studies included this aspect.^4758 60 66 69 71 73^,^75 79^ Young people’s reflections on health also included references to physical appearance, for example, being in good shape, having beautiful hair or ‘looking good’.^65 66 68 73 78 80 81^ Being overweight was seen as unhealthy,^47 65 69 70 78 80^ being underweight was identified as a negative health factor in homeless young people.^58^ Alcohol, tobacco and substance use were seen mostly as negative health factors^58 65 69 70 72 76 77 79^; in two studies,^71 73^ they were viewed as social tools. Also, medical aspects such as symptoms, absence of illness^4758 66 69 75 77 79^,^81^ and visualisations of organ systems^77^ were included in the conceptualisation of health. Three studies that included young people with chronic health conditions referred to medical treatments.^66 80 81^

##### Survey question: ‘How is your health in general’?

We identified two studies that investigated the conceptualisation of health in the survey context.^60 67^ When respondents are asked about their ‘health in general’, they consider symptoms, illnesses and fitness^60 67^; and physical activity and diet.^67^ The study by Joffer *et al*^67^ included information on the perception of different response options. *Very good* was interpreted by some respondents as feeling good most of the time. *Rather good* was preferred if *very good* was felt as an unattainable state, however, some respondents perceived it as having a negative undertone.^67^ *Neither good nor bad* was felt to be partly negative, while other respondents thought of it as a neutral state. *Rather bad* was perceived as an indication of having problems such as mental health issues; and *Very bad* was an extreme value, indicating serious conditions or events in the family.^67^

### Social dimension: relationships with friends, family and (in) school

All 21 studies included references to the social dimension of health. 13 studies identified supportive relationships with family and friends as a component of health.^5859 65 66 68^,^72 74 75 78^ One study discussed family as a source of conflict^76^ or violence,^75^ and how friends can facilitate the uptake of negative behaviours.^76^ Homeless young people mentioned family as a cause of homelessness and street friends as a supportive network.^58 79^ School as a positive working environment with supportive teachers and importance of education featured in eight studies.^47 65 67 68 71 76 78 81^ In two studies, school was felt to be a place that makes children happy.^69 73^ Seven studies discussed the school as a source of stress due to homework load, need to juggle school and work, negative experiences with teachers, or experienced bullying and difficulties.^60 67 68 70 73 75 76^

### Mental dimension: emotions and subjectivity of health

All 21 studies included elements from the mental dimension of health. 11 studies referred to positive emotions such as fun, excitement, happiness.^6668 69 71 73 75 77^,^81^ Good self-esteem and confidence were mentioned in five studies.^58 71 73 74 78^ Eight studies discussed how negative feelings such as anger, stress and tiredness have an impact on young people’s health.^5860 67^,^71 75^ The variety of emotions was summarised as ‘an emotional roller coaster’ in Larsson *et al.*^68^ Respondents in three studies perceived health as subjective and different for everyone.^59 69 79^

### Intersectionality of health and striving for balance

In 16 studies, respondents acknowledged that health develops in the intersectionality of different dimensions of health. Exceptions were five studies that had used data collected by visual methods or writings.^47 66 77 80 81^ In two studies, respondents discussed the relationship between mental and physical factors,^69 75^ or health as a holistic concept that includes elements from physical, mental and social dimensions.^58 74^ One study that included young people who had witnessed violence discussed how emotions impacted their physical health.^75^ One study highlighted how being unsupported and not doing well at school resulted in low self-esteem and loss of confidence.^73^ In Borraccino *et al,*^65^ financial assets were discussed alongside social relationships. Respondents in eight studies viewed balanced life and moderation as necessary for good health.^5859 68^,^74^ Here, striving for balance involves facing challenges that arise from an individual’s own goals,^68 71^ changing emotions,^68 71 73^ perceived expectations^59 69 70 74^ and physical growth: *It happens whether I like it or not, you have to try to adapt to it *^68^ (p76). Coping strategies include different things such as supportive relationships, having fun, experimenting with makeup, engaging in physical activity or a variety of material resources such as having one’s own bedroom.^67 68 70 71 73^ Young people in four studies viewed alcohol or substance use as part of social interaction.^58 71 73 79^ Healthy behaviours are thought to balance out the unhealthy ones.^59 69 74^

#### Survey question: ‘How do you feel’?

One study investigated the conceptualisation of health in the survey context when the general health question uses the word ‘feel’.^67^ When respondents discuss the question *A person may feel good sometimes and bad sometimes. How do you feel most of the time?,*^67^ they view health as a holistic concept with an emphasis on social and mental aspects. Factors included social relationships, girls had an emphasis on stressors such as homework, school achievement and expectations.^67^ In Välimaa,^60^ when respondents discussed aspects of health overall, they acknowledged the role of relationships, mental health, school attendance, healthy (home-cooked) food and clothing.

### Theme 2: health in context

Sixteen studies included references to the contextual factors.^5865 66 68^,^70 72 74^

#### Physical context: environment, housing and safety

The following environmental factors were mentioned as relevant to young people’s health: safe and green outdoor environment,^58 69 70 72 74 78^ cleanliness and pollution,^69 74^ lack of sidewalks or places that offer healthy food^70 74^ and noise.^78^ Two studies that included homeless young people discussed the lack of clean and affordable housing.^58 79^ It was interesting that also five studies that used drawings or writings included references (although brief) to contextual factors.^47 66 77 80 81^ Violence at home and in the context of war^58 75^ or colonialism^72^ was recognised as harming health.

#### Material resources

In five studies, young people talked about material resources such as individual possessions, own space, sufficient family income and good housing as important for health.^65 69 70 74 78^ Homeless young people discussed access to adequate clothing and healthy food.^58 79^ In the US context, the availability of health insurance is important.^70^ The availability of time to support healthy behaviours was discussed in two studies.^70 78^ In two studies, young people stated that financial aspects beyond basic needs were not important.^69 74^

#### Informational context and technology

Informational context and technology were referenced in seven studies.^58 59 70 73 76 78 80^ Images and health information in the media were perceived as challenging because of their unattainability.^73 78^ However, for some respondents, the same imagery had the potential to boost confidence.^78^ Too much screen time was perceived as unhealthy,^59 78^ but phones also provided a tool for staying in touch with others.^78 80^ Internet and social media were perceived as contradictory to Iranian culture.^76^ In three studies, young people perceived health education provided by schools as unhelpful and contradictory.^58 70 78^

#### Cultural environment: traditions, culture and religion

Two studies identified spiritual connection with elders, identity and cultural practices as important health factors.^72 76^ However, community, traditions and religion were also seen as restrictive for health.^76^ One study that focused on perceptions of health among millennials found that health was viewed as ‘customised’ according to factors such as one’s personality or context: ‘Everyone has a different way of living’ and so, ‘Different people need different things’^59^ (p5). For homeless young people, street culture means being disconnected from mainstream culture.^58 79^

## Discussion

**Figure 2:**
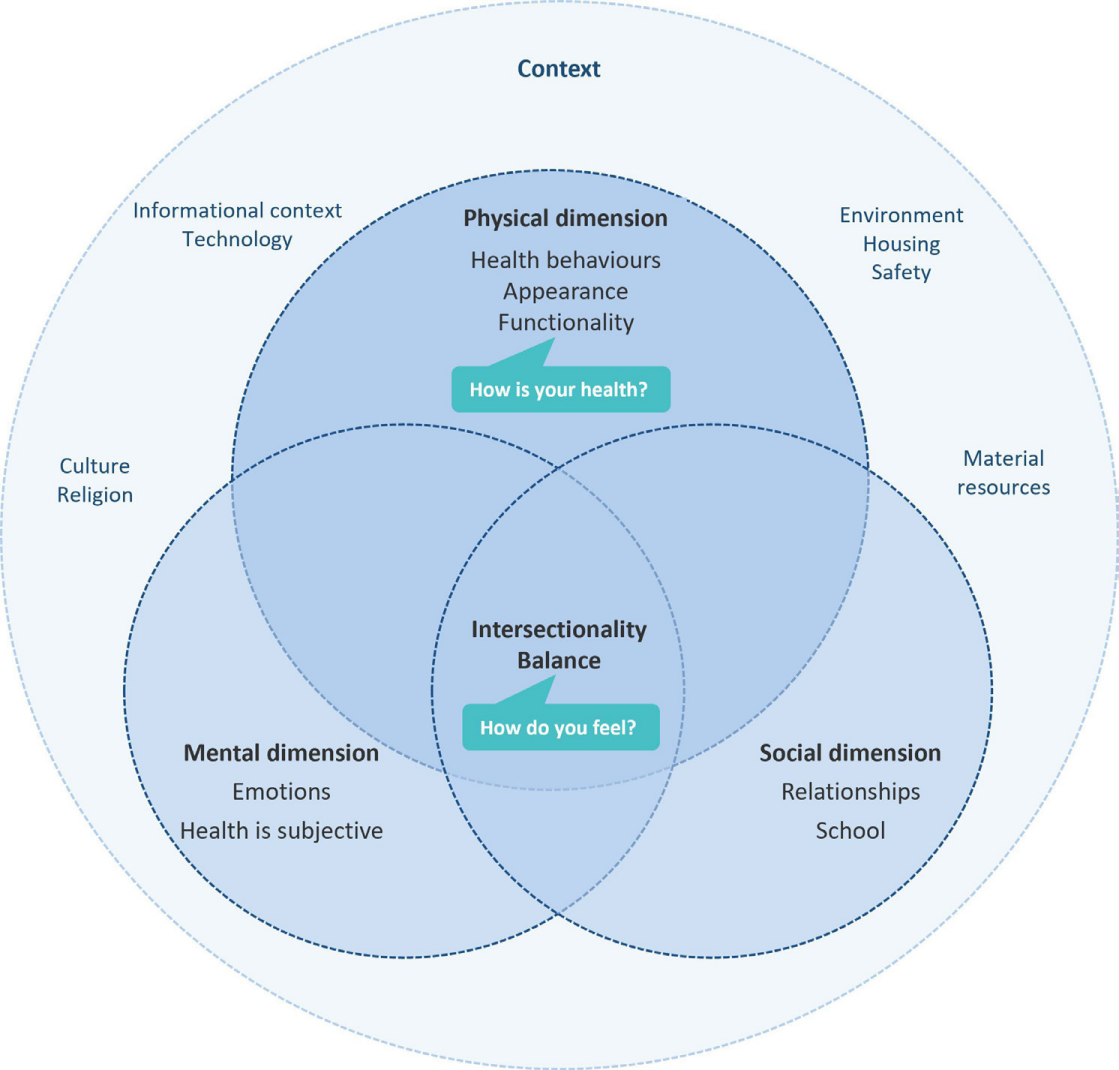
Analytical themes: dimensions of health and health in context.

This review aimed to synthesise the findings of the qualitative studies that investigate young people’s conceptualisation of health. Twenty-one studies were included in the synthesis. Our findings demonstrate that young people focus on physical factors of health when they respond to self-rated general health questions in the surveys. However, health overall is seen as an outcome of the interaction between physical, social and mental factors, which are situated within the broad context of young people’s lives. We developed two analytical themes and eight subthemes as visualised in figure 2.

We found that factors from the physical dimension of health dominate in young people’s conceptualisation of health. This can be because 18 included studies were completed in European or North American countries where school curricula and public health messages focus on health behaviours such as diet and physical activity.^47 67 69 78^ However, also studies completed in Turkey^77^ and India^47^ indicated that young people focus on physical factors when they talk about health. Indeed, in most countries globally, the school health policies have an emphasis on nutrition, physical education and substance use.^82^ We know that health education programmes influence young people’s understanding of health and can lead to the dominance of the ‘healthism’ discourse.^24 32 47 83^ Also, widespread social media use enforces similar messages with *looking good* as an indicator of good health being an important social marker, particularly among girls.^2932 44 83^,^85^

The importance of the structure in the formation of good health is seen in young people’s narratives about relationships with parents and teachers^23 35 36 38 86 87^; and contextual factors such as societal attitudes and material resources.^20 21 25 34 41 88^ These factors can be health-giving or health-harming, with the latter being acutely visible in the accounts of young people who experience homelessness, violence, difficulties at school or disadvantaged socioeconomic circumstances.^58 70 72 73 75 79^ As for broad contextual factors, religion and culture can be supportive of health,^22^,^2441^ or health-harming by imposing restrictions or subjecting young women to violence.^35 76^ Also, historical discrimination and racism are embedded in the health-harming structure. This can operate by exposing certain ethnic groups or refugees to poor living environments or racism, or through intergenerational trauma that has resulted in the adoption of harmful health behaviours as coping strategies.^41 72^ Therefore, the findings of this review support the understanding of health as an interaction between structure and agency situated within prevailing sociocultural and medical norms.^15 19 32 84^ We must remember that adolescence is a period of rapid biological and social changes and a time to acquire assets for later health.^50^ The combination of individual exposure to risk factors and the particularity of neurobiological development makes some young people more vulnerable to harmful behaviours and poor future health outcomes.^89^,^91^

Our findings also align with studies completed with adults and children and definitions of health such as Porta and Last^92^: *A structural, functional and emotional state that is compatible with effective life as an individual and as a member of society.* An exception was the study by Michaelson *et al*^59^ where themes focused on the ‘customisation’ and ‘subjectivity’ of health. This study used the generational theory that focuses on the culture of different generations and utilised focus group methodology, which facilitates the capturing of group norms.^93^ Perhaps one explanation for this could be found in the theory developed by Kuhn, which argues that science operates within shared frameworks of accepted theories and knowledge.^94^ Drawing on this argument, health education programmes have traditionally focused on individual health behaviours, and this would lead young people predominantly to consider physical factors when asked about their ‘health’. However, studies that used the broader term ‘feel’ in self-assessed health questions resulted in young people considering social and mental dimensions of health, and how they influence each other.

### Strengths and limitations

This review has the following strengths and limitations. We searched four databases that represent the major sources of medical, psychological and social science research. However, we agree that qualitative studies are hard to identify from database searches.^57^ This was mitigated by using a pearl-growing strategy based on three ‘gold standard papers’ and forward and backward citation searches.^14 57 65 67^ Second, included studies were restricted to those which involved 10–24-year olds. This restriction was applied to inform survey research that applies this common age group definition of young people. Third, we decided to focus on the conceptualisation of ‘health’ and exclude related terms such as ‘wellbeing’. Although a broad review that includes all related terms would have been desirable, we did not have the resources for doing this. In some cases, it was difficult to decide on the inclusion of studies where related terms were applied interchangeably. We mitigated by being transparent about this aspect and decided to include the potentially disputable studies in the supplemental file 5. Fourth, only two included studies investigated the conceptualisation of health in the survey context, with one including information on the perception of different health ratings. None of the included studies included respondents from lesbian, gay, bisexual and trans communities.

### Recommendations

We strongly recommend using reporting guidelines when reporting qualitative research. While all studies contributed towards understanding the conceptualisation of health, quality appraisal demonstrated that some aspects remained obscure on too many occasions. Future research should also address the gap in evidence on young people’s health evaluation process. It would be desirable to include related terms of well-being, mental health and quality of life. These terms are increasingly used in surveys, and interchangeably in research. Conceptual clarity would significantly enhance the interpretation and synthesis of research findings.

## Conclusion

To the best of our knowledge, this is the first systematic review that focuses on the conceptualisation of health among 10–24-year olds. Although health in the surveys is assessed based on physical measures, the perception of health develops in the wider context of young people’s lives, which contains both opportunities and risks. To understand different aspects of young people’s health, qualitative research must aim for conceptual clarity. This will improve the interpretation and synthesis of research findings that inform health-supporting strategies.

## Supplementary material

10.1136/bmjph-2024-001648 online supplemental file 1

## Data Availability

All data relevant to the study are included in the article or uploaded as supplementary information.
