# Supplementary material for: Conceptualisation of health among young people: a systematic review and thematic synthesis of qualitative studies
Source: BMJ Public Health. 2025 Jun 25;3(1):e001648. doi: 10.1136/bmjph-2024-001648 (PMC12198792; doi:10.1136/bmjph-2024-001648)
Supplement: online supplemental file 1 [file bmjph-3-1-s001.pdf]

SUPPLEMENTAL FILES

Conceptualisation of health among young people: a systematic review and thematic synthesis of qualitative studies

Katrin Metsis*, Joanna Inchley, Andrew James Williams, Sebastian Vrahimis, Lamorna Brown, Frank Sullivan

Supplemental file 1.

Enhancing transparency in reporting the synthesis of qualitative research (ENTREQ) checklist (1)

NO	Item	Guide and description	Page number
**1**	Aim	State the research question the synthesis addresses.	5
**2**	Synthesis methodology	Identify the synthesis methodology or theoretical framework which underpins the synthesis, and describe the rationale for choice of methodology *(e.g. meta-ethnography, thematic synthesis, critical interpretive synthesis, grounded theory synthesis, realist synthesis, meta-aggregation, meta-study, framework synthesis).*	7
**3**	Approach to searching	Indicate whether the search was pre-planned (*comprehensive search strategies to seek all available studies)* or iterative (*to seek all available concepts until they theoretical saturation is achieved*).	5
**4**	Inclusion criteria	Specify the inclusion/exclusion criteria *(e.g. in terms of population, language, year limits, type of publication, study type).*	5-6
**5**	Data sources	Describe the information sources used (e.g. *electronic databases (MEDLINE, EMBASE, CINAHL, psycINFO, Econlit), grey literature databases (digital thesis, policy reports), relevant organisational websites, experts, information specialists, generic web searches (Google Scholar) hand searching, reference lists)* and when the searches conducted; provide the rationale for using the data sources.	6
**6**	Electronic Search strategy	Describe the literature search *(e.g. provide electronic search strategies with population terms, clinical or health topic terms, experiential or social phenomena related terms, filters for qualitative research, and search limits)*.	Suppemental file 2
**7**	Study screening methods	Describe the process of study screening and sifting *(e.g. title, abstract and full text review, number of independent reviewers who screened studies).*	6
**8**	Study characteristics	Present the characteristics of the included studies *(e.g. year of publication, country, population, number of participants, data collection, methodology, analysis, research questions).*	10-12 Supplemental file 3

**9**	Study selection results	Identify the number of studies screened and provide reasons for study exclusion *(e,g, for comprehensive searching, provide numbers of studies screened and reasons for exclusion indicated in a figure/flowchart; for iterative searching describe reasons for study exclusion and inclusion based on modifications t the research question and/or contribution to theory development).*	8-9
**10**	Rationale for appraisal	Describe the rationale and approach used to appraise the included studies or selected findings *(e.g. assessment of conduct (validity and robustness), assessment of reporting (transparency), assessment of content and utility of the findings).*	7-8
**11**	Appraisal items	State the tools, frameworks and criteria used to appraise the studies or selected findings *(e.g. Existing tools: CASP, QARI, COREQ, Mays and Pope* [25]*; reviewer developed tools; describe the domains assessed: research team, study design, data analysis and interpretations, reporting).*	7-8, Supplemental file 4
**12**	Appraisal process	Indicate whether the appraisal was conducted independently by more than one reviewer and if consensus was required.	6-7, 10
**13**	Appraisal results	Present results of the quality assessment and indicate which articles, if any, were weighted/excluded based on the assessment and give the rationale.	6-7, 10
**14**	Data extraction	Indicate which sections of the primary studies were analysed and how were the data extracted from the primary studies? *(e.g. all text under the headings "results /conclusions" were extracted electronically and entered into a computer software).*	10
**15**	Software	State the computer software used, if any.	8
**16**	Number of reviewers	Identify who was involved in coding and analysis.	13
**17**	Coding	Describe the process for coding of data (e.g. line by line coding to search for concepts).	13
**18**	Study comparison	Describe how were comparisons made within and across studies *(e.g. subsequent studies were coded into pre-existing concepts, and new concepts were created when deemed necessary).*	10

**19**	Derivation of themes	Explain whether the process of deriving the themes or constructs was inductive or deductive.	7
**20**	Quotations	Provide quotations from the primary studies to illustrate themes/constructs, and identify whether the quotations were participant quotations of the author's interpretation.	15-17 (Box 2)
**21**	Synthesis output	Present rich, compelling and useful results that go beyond a summary of the primary studies (e.g. *new interpretation, models of evidence, conceptual models, analytical framework, development of a new theory or construct).*	15-20

PRISMA Checklist

Section and Topic	Item #	Checklist item	Location where item is reported
**TITLE**			
Title	1	Identify the report as a systematic review.	Title page line 1
**ABSTRACT**			
Abstract	2	See the PRISMA 2020 for Abstracts checklist.	Page 2
**INTRODUCTION**			
Rationale	3	Describe the rationale for the review in the context of existing knowledge.	Page 3-4
Objectives	4	Provide an explicit statement of the objective(s) or question(s) the review addresses.	Page 5
**METHODS**			
Eligibility criteria	5	Specify the inclusion and exclusion criteria for the review and how studies were grouped for the syntheses.	Page 5-6
Information sources	6	Specify all databases, registers, websites, organisations, reference lists and other sources searched or consulted to identify studies. Specify the date when each source was last searched or consulted.	Page 6
Search strategy	7	Present the full search strategies for all databases, registers and websites, including any filters and limits used.	Supplemental file 2
Selection process	8	Specify the methods used to decide whether a study met the inclusion criteria of the review, including how many reviewers screened each record and each report retrieved, whether they worked independently, and if applicable, details of automation tools used in the process.	Page 7
Data collection process	9	Specify the methods used to collect data from reports, including how many reviewers collected data from each report, whether they worked independently, any processes for obtaining or confirming data from study investigators, and if applicable, details of automation tools used in the process.	Page 7

Section and Topic	Item #	Checklist item	Location where item is reported
Data items	10a	List and define all outcomes for which data were sought. Specify whether all results that were compatible with each outcome domain in each study were sought (e.g. for all measures, time points, analyses), and if not, the methods used to decide which results to collect.	Page 7
	10b	List and define all other variables for which data were sought (e.g. participant and intervention characteristics, funding sources). Describe any assumptions made about any missing or unclear information.	Page 7
Study risk of bias assessment	11	Specify the methods used to assess risk of bias in the included studies, including details of the tool(s) used, how many reviewers assessed each study and whether they worked independently, and if applicable, details of automation tools used in the process.	Page 7-8
Effect measures	12	Specify for each outcome the effect measure(s) (e.g. risk ratio, mean difference) used in the synthesis or presentation of results.	NA
Synthesis methods	13a	Describe the processes used to decide which studies were eligible for each synthesis (e.g. tabulating the study intervention characteristics and comparing against the planned groups for each synthesis (item #5)).	NA
	13b	Describe any methods required to prepare the data for presentation or synthesis, such as handling of missing summary statistics, or data conversions.	NA
	13c	Describe any methods used to tabulate or visually display results of individual studies and syntheses.	Supplemental files 6 and 8
	13d	Describe any methods used to synthesize results and provide a rationale for the choice(s). If meta-analysis was performed, describe the model(s), method(s) to identify the presence and extent of statistical heterogeneity, and software package(s) used.	Page 8
	13e	Describe any methods used to explore possible causes of heterogeneity among study results (e.g. subgroup analysis, meta-regression).	NA
	13f	Describe any sensitivity analyses conducted to assess robustness of the synthesized results.	NA
Reporting bias assessment	14	Describe any methods used to assess risk of bias due to missing results in a synthesis (arising from reporting biases).	NA
Certainty assessment	15	Describe any methods used to assess certainty (or confidence) in the body of evidence for an outcome.	NA
**RESULTS**			
Study selection	16a	Describe the results of the search and selection process, from the number of records identified in the search to the number of studies included in the review, ideally using a flow diagram.	Page 8-9
	16b	Cite studies that might appear to meet the inclusion criteria, but which were excluded, and explain why they were excluded.	Page 9 and Supplemental file 5

Section and Topic	Item #	Checklist item	Location where item is reported
Study characteristics	17	Cite each included study and present its characteristics.	Page 10-12 and Supplemental file 3
Risk of bias in studies	18	Present assessments of risk of bias for each included study.	Page 13 Supplemental file 7
Results of individual studies	19	For all outcomes, present, for each study: (a) summary statistics for each group (where appropriate) and (b) an effect estimate and its precision (e.g. confidence/credible interval), ideally using structured tables or plots.	NA
Results of syntheses	20a	For each synthesis, briefly summarise the characteristics and risk of bias among contributing studies.	Page 13-14
	20b	Present results of all statistical syntheses conducted. If meta-analysis was done, present for each the summary estimate and its precision (e.g. confidence/credible interval) and measures of statistical heterogeneity. If comparing groups, describe the direction of the effect.	NA
	20c	Present results of all investigations of possible causes of heterogeneity among study results.	NA
	20d	Present results of all sensitivity analyses conducted to assess the robustness of the synthesized results.	NA
Reporting biases	21	Present assessments of risk of bias due to missing results (arising from reporting biases) for each synthesis assessed.	NA
Certainty of evidence	22	Present assessments of certainty (or confidence) in the body of evidence for each outcome assessed.	NA
**DISCUSSION**			
Discussion	23a	Provide a general interpretation of the results in the context of other evidence.	Page 20-23
	23b	Discuss any limitations of the evidence included in the review.	Page 22
	23c	Discuss any limitations of the review processes used.	Page 22
	23d	Discuss implications of the results for practice, policy, and future research.	Page 23
**OTHER INFORMATION**			
Registration and protocol	24a	Provide registration information for the review, including register name and registration number, or state that the review was not registered.	Page 3
	24b	Indicate where the review protocol can be accessed, or state that a protocol was not prepared.	Page 3 and 5
	24c	Describe and explain any amendments to information provided at registration or in the protocol.	Page 25
Support	25	Describe sources of financial or non-financial support for the review, and the role of the funders or sponsors in the review.	Page 24
Competing interests	26	Declare any competing interests of review authors.	Page 24

Section and Topic	Item #	Checklist item	Location where item is reported
Availability of data, code and other materials	27	Report which of the following are publicly available and where they can be found: template data collection forms; data extracted from included studies; data used for all analyses; analytic code; any other materials used in the review.	Manuscript

From: Page MJ, McKenzie JE, Bossuyt PM, Boutron I, Hoffmann TC, Mulrow CD, et al. The PRISMA 2020 statement: an updated guideline for reporting systematic reviews. BMJ 2021;372:n71. doi: 10.1136/bmj.n71. This work is licensed under CC BY 4.0. To view a copy of this license, visit https://creativecommons.org/licenses/by/4.0/

Supplemental file 2. Search strategy

1) MEDLINE (Ovid®)

Preliminary strategy in January 2022. Updated and searched in March 2023 and 24.04.2024

1. Adolescent/ or Adolescent Health/ or Adolescent Medicine/ or Young Adult/
2. (adolescen* or youth* or young or young people or young adult or teen*).mp.
3. 1 or 2
4. "surveys and questionnaires"/ or health surveys/ or patient health questionnaire/ or exp self report/
5. (Self-assessed health or Self-rated health or Perceived health or Self-evaluated health or Self-reported health or Self-ratings of health or Self-assessments of health or Self-perceptions of health or Self-evaluations of health or Self-evaluation of health).mp.
6. ((subjective or self-report* or self-assess* or self-rat* or perce* or self-evaluat*) adj5 health).mp.
7. Health Status/
8. 4 or 5 or 6 or 7
9. 3 and 8
10. exp thinking/ or exp concept formation/ or judgment/
11. exp diagnostic self evaluation/
12. exp Comprehension/cl, ph [Classification, Physiology]
13. Attitude to Health/eh, sn [Ethnology, Statistics & Numerical Data]
14. ((Conceptualis* or perce* or perception or view or feel or subjective) adj5 health).mp.
15. 10 or 11 or 12 or 13 or 14
16. 9 and 15
17. qualitative.mp.
18. 16 and 17
19. limit 18 to structured abstracts

2) **ProQuest Sociology Collection (Applied Social Sciences Index & Abstracts (ASSIA) / Sociological Abstracts / Sociology Database**

String 1 (June 2022, updated March 2023)

(MAINSUBJECT.EXACT("Adolescents") OR (MAINSUBJECT.EXACT("Students") OR MAINSUBJECT.EXACT("Pupils")) OR (MAINSUBJECT.EXACT("Young adults") OR MAINSUBJECT.EXACT("Young people") OR MAINSUBJECT.EXACT("Young adulthood"))) AND (ab(conceptualization) AND ab(health))

String 2 (June 2022, updated March 2023)

((MAINSUBJECT.EXACT("Health status") OR MAINSUBJECT.EXACT("Health")) OR (MAINSUBJECT.EXACT("Selfappraisal") OR MAINSUBJECT.EXACT("Selfassessment"))) AND (MAINSUBJECT.EXACT("Meaning") OR MAINSUBJECT.EXACT("Conceptualization")) AND (MAINSUBJECT.EXACT("Adolescents") OR (MAINSUBJECT.EXACT("Students") OR MAINSUBJECT.EXACT("Pupils")) OR (MAINSUBJECT.EXACT("Young adults") OR MAINSUBJECT.EXACT("Young people") OR MAINSUBJECT.EXACT("Young adulthood"))) AND pd(20220101-20230317)

String 3 (June 2022, deemed faulty in March 2023)

((if(health perspectives) OR if(health)) AND (if(young people) OR if(Youth))) AND (((MAINSUBJECT.EXACT("Health status") OR MAINSUBJECT.EXACT("Health")) OR (MAINSUBJECT.EXACT("Selfappraisal") OR MAINSUBJECT.EXACT("Selfassessment"))) AND (MAINSUBJECT.EXACT("Adolescents") OR (MAINSUBJECT.EXACT("Students") OR MAINSUBJECT.EXACT("Pupils")) OR (MAINSUBJECT.EXACT("Young adults") OR MAINSUBJECT.EXACT("Young people") OR MAINSUBJECT.EXACT("Young adulthood")))) AND pd(20220101-20230317)

String 4 (March 2023)

(mainsubject(health) AND (mainsubject(selfappraisal) OR mainsubject(selfassessment)) AND (mainsubject(students) OR mainsubject(pupils) OR mainsubject(young adults) OR mainsubject(young people) OR mainsubject(adolescents))) OR (((IF(health) OR IF(health perspectives)) AND (IF(young people) OR IF(young adulthood) OR IF(youth) OR mainsubject.Exact("adolescence/adolescent/adolescents"))) AND abstract(qualitative))

3) PsycINFO (APA PsycNet)

(((((title:(adolescen*))) OR ((title:(young))) OR ((title:(child*))) OR ((title:(youth)))) AND ((((((title:(health*)))) AND ((((((((IndexTerms:(adolescent attitudes)) OR (IndexTerms:(Happiness)) OR (IndexTerms:(health behavior)) OR (IndexTerms:(well being)) OR (Index Terms:(word meaning)) OR (Index Terms: (Health Status)) OR (Index Terms: (Health)) OR (Index Terms: (Self-Evaluation)))))) OR (IndexTerms:(adolescent health))) AND (((AgeGroupFilt:("Childhood (birth-12 yrs)") OR AgeGroupFilt:("School Age (6-12 yrs)") OR AgeGroupFilt:("Adolescence (13-17 yrs)") OR AgeGroupFilt:("Young Adulthood (18-29 yrs)"))))))) AND (((MethodologyFilt:("Interview") OR MethodologyFilt:("Qualitative Study"))))) *NOT* Title: sex* *NOT* Title: reproduct* *NOT* Title: nurse* *NOT* Title: intervention* *NOT* Keywords: "substance use" *NOT* Keywords: bully* *AND* Population Group: Human *OR* Female *OR* Male *AND* Peer-Reviewed Journals only

4) Web of Science Core Collection™

(AB=("self-rated health" OR "subjective health" OR "self-assessed health" OR "self-reported health" OR "general health" OR "global health" OR healthy) AND AB=(adolescen* OR youth OR "young people" OR teen*) AND AB=(concept* OR meaning OR mean* OR definition OR perspective* OR explanation* OR perception* OR construction OR experience* OR interpret* OR reason*) AND AB=(interview* OR qualitative OR "qualitative research" OR "focus groups" OR "think-aloud") AND TI=(health*))

Supplemental file 3. Criteria used to appraise study quality, a modified version of the Quality Framework (2)

Section	The Quality Framework questions
Findings	1. How credible are the findings?
	2. How has knowledge/ understanding been extended by the research?
	3. How well does the study address its original aims and purpose?
	4. Scope for drawing wider inference – how well is this explained?
Design	5. How defensible is the research design?
Sample	6. How well defended is the sample design/ target selection of cases/ documents?
	7. Sample composition/ case inclusion – how well is the eventual coverage described?
Data collection	8. How well was the data collection carried out?
Analysis	9. How well has the approach to, and formulation of, the analysis been conveyed?
	10. Contexts of data sources - how well are they retained and portrayed?
	11. How well has diversity of perspective and content been explored?
	12. How well has detail, depth and complexity (i.e., richness) of the data been conveyed?)
Reporting	13. How clear are the links between data, interpretation and conclusions – i.e., how well can the route to any conclusions be seen?
	14. How clear and coherent is the reporting?
Reflexivity and neutrality	15. How clear are the assumptions /theoretical perspectives/values that have shaped the form and output of the study?
Ethics	16. What evidence is there of attention to ethical issues?
Auditability	17. How adequately has the research process been documented?

Supplemental file 4. Excluded studies identified from database searches, reference lists or citations.

Exclusion reasons: broader age range, concept of health not the focus of the study, insufficient data for synthesis, health in the context of certain phenomena such as diet or physical activity.

Reference and country	Background and aims	Age	Database search	Ref list or citation
Aho et al 2016 **(3)**<br>Sweden	To describe health perceptions related to the sense of coherence among young adults living with recessive limb-girdle muscular dystrophy.	18-30	x	
Barco Leme et al. 2021 **(4)**<br>The US	Volunteer database, aged 10-14, no health conditions that affect diet. Definitions of health, nutrition, and food groups. Health ratings.	10-14	x	
Buck and Ryan-Wenger 2003 **(5)**<br>The US	Low-income inner-city middle school.<br>To describe the meaning of health in early adolescence and construct a taxonomy of health concepts.	10-14		x
Burrows et al 2009 **(6)**<br>New Zealand	To investigate New Zealand children's understanding of 'health'.	8-9<br>12-13	x	
Burrows et al 2002 **(7)**<br>New Zealand	Explore the meanings of health and fitness.	8-9<br>12-13		x
Caluzzi et al 2021 **(8)**<br>Australia	Light drinkers and abstainers. Themes explored: leisure activities, school, family, social groups, technology, health, and perceptions of alcohol.	16-19	x	
Cronely et al 2019 **(9)**<br>The US	Perceptions of health and nature among adolescents living in emergency family shelters.	13-17	x	
De Moura et al 2003 **(10)**<br>Brazil	Groups of deprived, privileged and street children. Definition of health: What is health? Definition of illness: What is illness?	Mean 15-16	x	
Dixit et al 2018 **(11)**<br>The US	High school. To identify adolescent Burmese refugee perspectives on determinants of health and health-related experiences.	Grades 10 and 12	x	
Friderichs MS. 2018 **(12)**<br>Australia	To explore how Indigenous young women in Katherine think about health, experience it in everyday life, and interact with the health system and social services.	16-24		x
Glozah FN. 2015 **(13)**<br>Ghana	Senior high schools.<br>How perceived social support and stress influence the construction of the meaning of health and wellbeing to Ghanaian adolescents.	Mean 16.86	x	
Harris et al 2018 **(14)**<br>UK	State secondary schools across England.<br>Explore young people's knowledge and understanding of health, fitness, and physical activity.	12-15<br>Adults	x	
Hinton and Earnest 2009 **(15)**<br>Papua New Guinea	Rural women.<br>Examine young women's perceptions of health and health-related experiences.	15-29	x	
Hobin and Anderson 2008 **(16)**	One class in public school (Canada and BVI).<br>Cross-cultural research.	Means:<br>11.2<br>10.5		x

Canada and the British Virgin Islands (BVI)	To explore middle-school students' concepts of health.			
Hsin et al 2020 **(17)** Taiwan	Students from three educational stages. Cross-sectional survey to address broader, varied, and developmental health concepts.	13±0.6, 16±0.6, 19±0.6	Google	
Isaak and Marchessault 2008 **(18)** Canada	To explore perspectives on the meaning of the health of Aboriginal adults and youth Manitoba First Nations community.	12-19 Adults	x	
Isma et al 2023 **(19)** Sweden	Investigate the perceptions of health among school-aged children and youth leaders from socially vulnerable areas.	10-12 Adults	x	
Kroh et al 2023 **(20)** Germany	How do children rate their health according to this question "In general, how would you rate your health?"	9-12		x
Kefford et al 2005 **(21)** Australia	To identify perceptions of health, health concerns, and health service needs among young people in a suburb of Sydney, New South Wales.	14-24	x	
Martin et al 2018 **(22)** Ireland	To explore children's and young people's understanding of health and factors that facilitate or hinder healthy lifestyles	7-17	x	
McCloughen et al 2016 **(23)** Australia	Young people experiencing mental illness and taking psychotropic medication. To explore understandings that influence behaviours related to physical health and wellbeing.	16-25	x	
Millstein and Irwin 1987 **(24)** The US	Adolescents from inner-city school district. To examine adolescents' concepts of health and illness simultaneously.	11-18		x
Natapoff JN. 1978 **(25)** The US	Three elementary and one junior high schools. To examine ideas which contribute to a concept of health and how these change as children develop.	Mean age 6.58-12.66		x
Pang et al 2016 **(26)** Australia	How young Chinese Australians understand health and (un)healthy bodies.	10-15	x	
Pfister et al 2017 **(27)** Denmark	Danish women, four high schools in Copenhagen. How women position themselves in the dominant discourses of health, exercise, and appearance.	16-20	x	
Piko and Bak 2006 **(28)** Hungary	Two schools from small towns. Describe 8–11-year olds' beliefs of health, illness, health promotion and disease prevention.	8-11		x
Quarmby T. 2013 **(29)** UK	How the family composition affects young people's understanding of health and dispositions.	11-14	x	
Ravenell et al 2006 **(30)** The US	Different groups: trauma survivors, HIV-positives, community etc. The aim was to identify and explore African American men's perceptions of health.	Adults and adolescents	x	
Secor-Turner et al 2016 **(31)** Kenya	What are the perceived barriers and facilitators of health for rural Kenyan adolescents?	12-26	x	
Singletary et al 2015 **(32)** UK	Interactive workshop about healthy eating, physical activity, and mental health. To explore the perceptions of health and wellbeing.	13-14		x
Sundar et al 2024 **(33)** Norway	To investigate the public health nursing students' and pre-service teacher students' perceptions of good or poor health.	20-45	x	

Zullig et al 2005 **(34)**<br>The US	To explore adolescent the relationships between adolescent self-rated health, physical health, mental health, and quality of life. Southern and Midwestern sample, high school students.	Grades 9-12 in southern sample.	x	
Van der Meer et al 2023 **(35)**<br>Germany	To explore concepts of (mental) health and illness of refugee youth as well as assess their mental health literacy.	11-21	x	
Velardo and Drummond 2019 **(36)**<br>Australia	To explore preadolescent children's perceptions of health and nutrition, and interrelated issues of nutrition, physical activity, and fatness.	11-12	x	
Walker et al 2019 **(37)**<br>Australia	What it means to young Indigenous people to be healthy and how social media influences health behaviours. Discussions informed by the Integrated Model of Behaviour.	17-24	x	
Wang W. 2004 **(38)**<br>China, Shanghai	Two primary and high schools, and two universities. To describe and explain the concept of health.	9-20		x
Woodgate and Skarlato 2015 **(39)**<br>Canada	To examine youth's perspectives of the relationships between health and environment.	12-19	x	
Wright and Burrows 2004 **(40)**<br>New Zealand	New Zealand's National Education Monitoring Project. To explore the meanings of being healthy and the key components of a healthy person.	8-9<br>12-13		x
Wright et al 2006 **(41)**<br>Australia	The Life Activity Project: the place and meaning of physical activity in young people's lives. Meanings of health and fitness.	15-18		x
Yu et al 2019 **(42)**<br>China, Shanghai	The Well-being of Adolescents in Vulnerable Environments (WAVE) global study. How internal migrant young people view the health issues, and the services and opportunities they could seek in their host community.	15-19<br>Adults	x	

Supplemental file 5. Characteristics of included studies

Reference	Country	Aim	Sample	SRH/health question(s)	Data collection method	Data analysis method	Key findings
		Studies that investigated the concept of health in the survey context; study question includes the word "health" or "feel"					
Joffer et al 2016 **(43)**	Sweden	How adolescents interpret and reason when answering a question on self-rated health (SRH).	N=58<br>Age: 12-13 and 17-18<br>Boys: N=29<br>Girls: N=29	A person may feel good sometimes and bad sometimes. How do you feel most of the time? Very good, Rather good, Neither good nor bad, Rather bad, Very bad.<br><br>Next, respondents were asked how they would have answered if asked about their "health".	Inductive research design 'Think-aloud' interviews	Qualitative content analysis of four groups:<br>Younger girls<br>Older girls<br>Younger boys<br>Older boys	• 'Feel' in SRH question: holistic and mental concept<br>• 'Health' in SRH question: health behaviours and physical aspects<br>Age and gender differences<br>Response options reflect differences in subjective health
Välimaa, R. 2000 **(44)**	Finland	To examine perceived health among 11-15-year-olds, and associations of different dimensions of health with SRH.<br>To investigate social networks and the association of social support with perceived health among 15-year-olds.	N=27<br>Boys: N=15<br>Girls: N=12<br>Age: 15 (9th grade pupils)	Questionnaire question[1]:<br>How healthy do you think you are? Very healthy, quite healthy (in some questionnaires, somewhat healthy), or not very healthy?<br><br>Focus group question[2]:<br>What did you think (when answering the	Focus group discussions	Qualitative content analysis<br>Observations	Health was mostly defined as physical strength, fitness, and absence of illness<br>Core themes<br>• Conceptualisation of health: "Are you properly fit?"<br>• Discussion about (subjective) symptoms: " … then

[1] *Question in Finnish: "Kuinka terveeksi koet itsesi? Erittäin terveeksi, melko terveeksi, en kovin terveeksi?"*
[2] *Question in Finnish: "Mitä te ajattelitte (vastatessanne kysymykseen), että mitä se terveydentila on?"*

Reference	Country	Aim	Sample	SRH/health question(s)	Data collection method	Data analysis method	Key findings
		How do 15-year-old adolescents understand the concept of health in the survey context?		question), what does that state of health mean?			the first symptoms start.”<br>• Factors influencing health: “Health is multifactorial.”<br>• Peer discussions about health: “Yeah we talk.”<br>Gender differences
Studies that investigated the concept of health generally; study question includes the word “health”							
Berman, H. 1999 **(45)**	Canada	How is health understood and experienced by two groups who have grown up amid violence, children of war and children of battered women?	Purposive sample<br>Age 10-17<br>Children of war<br>Males: N=5<br>Females: N=11<br>Children of battered women<br>Males: N=7<br>Females: N=9	What does health mean to you?	Critical narrative study<br>Individual and group interviews	Narrative and content analysis<br>NUD*IST software<br>Discussed emerging themes with children.	Themes:<br>• Not Being Sick<br>• Being Able to Do What You Want to Do<br>• Being Mentally Healthy and Happy and Stuff Like That<br>• Just Getting Through the Day
Borraccino, A., Pera, R., and Lemma P. 2019 **(46)**	Italy	To explore the core categories evoked when adolescents describe what it means to be ‘*healthy*’ and ‘*unhealthy*’.	Convenience sample, three different upper secondary schools<br>N=34<br>Age: mean 15,3<br>Male: N=19	Discussion of the features of ‘healthy’ and ‘unhealthy’ adolescents during the collage creation.	Conceptual projective techniques:<br>Collage creation<br>Group discussions using the think-aloud technique	Content analysis of group discussions	Core categories of healthy or unhealthy adolescents:<br>• Physical appearance<br>• Personal commitment and goals

Reference	Country	Aim	Sample	SRH/health question(s)	Data collection method	Data analysis method	Key findings
			Female: N=15				• Ownership of possessions and space<br>• Use of free time<br>• Social belonging
Cetin et al 2012 **(47)**	Turkey	Determine the views of ninth-grade students about the health concept.	N=156<br>9th grade students (age not specified, vocational schools)<br>Male N=81<br>Female N=75	Instruction to students: What is health? Explain and draw.	Qualitative approach<br>Drawing and writing technique	Content analysis	Themes:<br>• Definition of health concept<br>• Factors protecting health<br>• Factors affecting health negatively<br>• Human and human body<br>• Medicine<br>• Nutrition and nourishment
Dow et al 2022 **(48)**	Ireland	How girls from disadvantaged communities make sense of 'being healthy'.	N=22<br>Age: 10–12<br>Girls only	Conceptualisation of 'Being healthy' questions part of the longer topic guide.<br>Examples of prompts:<br>What does 'being healthy' mean?<br>How did you come up with that?<br>What does a healthy person look like?	Three focus groups<br>Pictures of celebrities followed by the questions from the topic guide	Phenomenological approach<br>Thematic analysis<br>Socioecological model	Themes:<br>• Looks tell all<br>• Health literacy: Salads, gym, sleep, repeat<br>• Being healthy with my friends<br>• Family as role models<br>• Our neighbourhood is unhealthy<br>• We learned it at school

Reference	Country	Aim	Sample	SRH/health question(s)	Data collection method	Data analysis method	Key findings
				What does an unhealthy person look like?			• Social media and health<br>• Overarching theme: Tendency for contradiction
Flick, U. and Röhnsch G. 2007 **(49)**	Germany	Which representations of health do homeless adolescents hold? Which forms of health practices are reported or can be observed?	N=24<br>Age: 14-20<br>Male N=12<br>Female N=12	The adolescents were asked for their concept of health, experiences of health, health problems and how they deal with them.	Social representations theory<br>Episodic interviews<br>Participant observation	Thematic coding	Definitions of health:<br>• Health as physical and mental well-being<br>• Health as a result of specific practices<br>• Health as absence of illness<br>• Health as functionality<br>Links between definitions and experiences of health:<br>• Health as a distant ideal<br>• Denial of health problems<br>• Health as relative<br>• Health as reality to a large extent<br>Meaningfulness of health and ways to influence it:<br>• Health as irrelevant

Reference	Country	Aim	Sample	SRH/health question(s)	Data collection method	Data analysis method	Key findings
							• Health as an option for activity
Hager, M. 1997 **(50)**	The US	What is the concept of health among adolescents with Insulin Dependent Diabetes Mellitus in a rural state?	Convenience sample<br>Diagnosed with insulin-dependent diabetes<br>N=8<br>Age: 12-18<br>Males: N=7<br>Females: N=1	Instructions<br>Photos: Use the camera to take pictures that show health.<br>Written description: Use the 'Record' section of these instructions to record your pictures and how they demonstrate health. Health is whatever you think it is. There is no right or wrong answer.<br>Interview: Tell me how the pictures show health?	Replication of a study by Hanna, Jacobs & Guthrie (1995)<br>Exploratory-descriptive study<br>Qualitative approach<br>Photography<br>Written descriptions of photos<br>Semi-structured interviews	Inductive content analysis<br>Pictures n=46<br>Descriptions n=80	• What health is:<br>Opposite of poor health<br>Signs of Health<br>Strength<br>Abilities and Functioning<br>Vitality – Energy and Excitement<br>• What one does to be healthy:<br>Nutrition<br>Exercise<br>Excitement /Fun/Dreams<br>Health Care<br>Social Support<br>Hygiene and Clothing<br>Environment
Hanna, K.M. and Jacobs, P. 1993 **(51)**	The US	1) What is the meaning of health among adolescents diagnosed with cancer?<br>2)How is the meaning of health communicated with the use of photography?	N=4<br>Age: 14-17<br>Male: N=3<br>Female: N=1	Writing instruction: "Describe a situation in which you experienced health. Share your thoughts, perceptions, and feelings about the situation."	Exploratory-descriptive pilot study:<br>Writing<br>Photography<br>Semi-structured interviews	Latent content analysis	• What health is:<br>Growing<br>Having a positive appearance<br>Being active<br>Having abilities<br>Being normal<br>Having feelings

Reference	Country	Aim	Sample	SRH/health question(s)	Data collection method	Data analysis method	Key findings
				Polaroid camera: "Use the camera to take pictures of situations of health."			Having a future.<br>• What one does to be healthy:<br>Diet and nutrition<br>Rest and exercise<br>Relationships<br>Resources
Hanna, K.M., Jacobs. P.M. and Guthrie, D. 1995<br>**(52)**	The US	To explore and describe the concept of health held by adolescents with diabetes.	Convenience sample<br>N=9<br>Age:12-19<br>Males: N=6<br>Females: N=3	Instruction to participants: to take pictures that demonstrate health and write briefly how the picture demonstrates health.	Exploratory study:<br>Photography (Polaroid cameras)<br>Written description of photos.	N=46 pictures<br>N=53 descriptions of health<br>Content analysis of written descriptions<br>Inductive generation of themes	• What health is:<br>Energy<br>Positive feelings<br>Absence of illness<br>Strength<br>Activity<br>Fitness<br>Mental abilities and functioning<br>Relationships<br>• What one does to be healthy:<br>Nutrition<br>Exercising<br>Diabetes care<br>Sleeping<br>Studying<br>Relaxing and recreational activties<br>Good hygiene<br>Obtaining shelter

Reference	Country	Aim	Sample	SRH/health question(s)	Data collection method	Data analysis method	Key findings
Hariharan et al 2019 **(53)**	India	How do school children conceptualise health? Does the concept of health show a progressive change across the age?	Purposive sampling<br>N=667<br>Age: 11-16<br>Male: 56%<br>Female: 44%	What do you understand by 'being healthy'?	Qualitative framework<br>Single open-ended question with the write in answer	Thematic approach, content analysis	Themes:<br>• Meaning of health<br>• Ways to be healthy<br>• Indices of health<br><br>Age differences
Karabanow et al 2007 **(54)**	Canada	How street youth understand health and wellness, how they define good and bad health, and their experiences in accessing diverse health services.	Pilot study<br>Convenience and purposive sampling<br>N=15<br>Age 16-24<br>Males N=12<br>Females N=3<br>N=10 health and social service providers	Number of different queries<br>Relevant to the conceptualisation of health:<br>What are street youth's perceptions, experiences and understanding of health and wellness?	Semi-structured, in-depth interviews<br>Survey to describe the sample	Interpretive or constructivist grounded theory approach	Key issues:<br>• What is "Health" Anyway?<br>• The Good and Bad of Street life<br>Freedom and anxiety of being on your own<br>Harassment from others<br>Travelling<br>A sense of belonging<br>• Daily Routines<br>• Health Risks on the Street<br>Mental health issues<br>Disconnected from others<br>• Health Seeking Behaviours<br>• So What's Needed?
Michaelson et al 2016 **(55)**	Canada	What are adolescent perceptions of health?	Purposive snowball sampling	Open-ended questions not specified – began with a general interest	Sequential mixed methods design	Generational theory	Three overarching themes:

Reference	Country	Aim	Sample	SRH/health question(s)	Data collection method	Data analysis method	Key findings
			N=40<br>Males: N=13<br>Females: N=27<br>Age: 12-15	in perceptions of health followed by an iterative strategy.<br>Discussion of definitions of health.	Core qualitative study: focus groups and photo elicitation	Qualitative constant comparative method of grounded theory	• Good health is customized: 'Health is different for everyone'<br>• Good health is subjective: 'The state that you want to be in'<br>• Good health is not absolute: 'You can be healthy without being completely healthy'
O'Higgins, S., Sixsmith, J. and Gabhainn S.N. 2010 **(56)**	Ireland	What adolescents understand by words of 'health' and 'happy'?	N=31<br>Age: 13<br>Males: N=16<br>Females: N=15	16 open-ended questions (8 for health and 8 for 'happy').<br>Health:<br>What makes you healthy?<br>What influences how healthy you are?<br>What will you do to make yourself healthy after you leave school?<br>When someone says the word health what do you think it means?<br>How would you describe being healthy?	Exploratory study<br>Grounded theory<br>Semi-structured interviews	Thematic content analysis<br>NUD*IST 5	Concept of health themes:<br>• Holistic views<br>• Health as a resource<br>• Subjective measures<br>• Illness was described as the opposite of being healthy<br>• Aetiology of health<br>• Body image<br>• Outside influences<br>• In the future<br><br>Gender differences

Reference	Country	Aim	Sample	SRH/health question(s)	Data collection method	Data analysis method	Key findings
				Do you think it is the same for everyone? Would someone in a wheelchair be healthy? How would you know that someone else is healthy?			
Ott et al 2011 **(57)**	The US	To describe adolescents' 'emic' views of health and provide implications for state policy.	Purposive sampling N=68<br>Age: 15-24<br>Male: N=34<br>Female: N=34	What makes a teen healthy?	Focus groups	A two-stage technique for identifying shared concepts and creating models of social cognitions held by social groups.	Conceptual model, 3 levels of health<br>• Individual-level factors: Obesity, stress and fatigue, alcohol, tobacco, and substance use, sexual behaviors, sexually transmitted infections, HIV, and adolescent pregnancy, violence and personal safety.<br>• Relationships: Supportive relationships with family, school and community.<br>• Environment and contexts: Physical, financial, and informational environments.

Reference	Country	Aim	Sample	SRH/health question(s)	Data collection method	Data analysis method	Key findings
Parvizy, S., Ahmadi, F. and Nasrabad, A.N. 2008 **(58)**	Islamic Republic of Iran	To gain an understanding of adolescents' perspectives on health and develop a categorical model of health.	N=52<br>Age: 11-19<br>Males: N=26<br>Females: N=26	How do you view your health as an adolescent?<br>What factors affect adolescents' health?<br>Who is a healthy adolescent?	Grounded theory<br>Semi-structured interviews	Open coding<br>Axial coding<br>Selective coding<br>Constant comparison analysis and theoretical sampling	Five concepts and the core variable:<br>• Community: contrast and/or obedience<br>• Friendship and relationships: to be accepted or to be dependent<br>• Education: worries and hopes<br>• Family: individuation or nurture<br>• Lack of limitation: trial and error<br>• Core variable: identity formation and emergence of identity
Randell et al 2016 **(59)**	Sweden	How adolescent boys understand the concept of health and what they find important for its achievement.	Purposive sampling<br>N=33<br>Age: 16-17	What does the word health mean to you?<br>What affects your health?<br>What do you do in order to feel well?<br>How do you deal with your health?	Interviews	Constructivist grounded theory approach.	• The emotional and relational mind: Health as an emotional experience: positive emotions, self-esteem, balance in life<br>Health as a relational experience: trustful relationships, sense of belonging<br>• The functional body: Doing health as a functional condition: body as a tool,

Reference	Country	Aim	Sample	SRH/health question(s)	Data collection method	Data analysis method	Key findings
							energy, and condition
Sasakamoose et al. 2016 **(60)**	Canada	To explore the understandings of health among First Nations and Metis youth and the health of their communities.	N=13<br>Age: 14-17	What does health look like to you?<br>What does health look like in your community?	Relational Indigenous epistemology<br>Decolonizing approach<br>Participatory Action Research<br>Sharing circles	Thematic analysis	Themes:<br>• Holistic health<br>• Culture (spirituality, identity, traditions)<br>• Sports and Well-Being<br>• Navigating Addictions
Woodgate et al. 2010 **(61)**	Canada	How youth define health in the context of their life situations: how youth define health, what it means to be healthy, how life situations influence their health.	Purposive and snowball sampling<br><br>N=71<br>Age: 12-19<br>Males: N=29<br>Females: N=42<br>Different ethnic groups<br>Lower-income neighbourhood	Questions to draw out views of health and individual and systemic influences related to health within life situations.	Open-ended person-centered interviews<br>Second interview supplemented by photovoice<br>Fieldwork including passive observation and participation in activities, fieldnotes	Ethnographic approach.<br>Analysis concurrent with data collection.<br>Recruitment ended when saturation was achieved.	Six sociocultural themes:<br>• There are many different types of health<br>• Health is the act of doing and not a state of being<br>• Personal lifestyle practices as the main determinants of health<br>• Beyond the talk of health<br>It is all about family and friends<br>Desire for a safe, clean, green, *and livable space)*<br>• Missing connections to the broader social

Reference	Country	Aim	Sample	SRH/health question(s)	Data collection method	Data analysis method	Key findings
							determinants and health<br>• It's really up to the kid that's doing it
Studies that investigated the concept of health generally; study question includes the word "feel"							
Larsson, M., Sundler, A.J. and Ekebergh, M. **(62)**	Sweden	Describe the phenomenon of health as experienced by adolescent girls in Sweden.	Convenience sample<br>N=15<br>Age: 13-19	Respondents were asked to express lived experiences of what made them feel good in everyday life and describe experiences that affected their health and well-being.	Reflective lifeworld approach<br>Phenomenological interviews<br>Photocards	Phenomenological analysis: describe the essence and constituents of the phenomenon	Constituents of health:<br>• The importance of togetherness<br>• To manage and incorporate demands and expectations—a tension between inadequacy and a zest for life<br>• An emotional roller-coaster<br>• To strive for balance
Spencer, G. 2013 **(63)**	England	To elicit young people's own understandings of health and enquire how these meanings may be shaped within the context of everyday lives.	Purposive sample<br>Age 15-16<br>Males: N=29<br>Females: N=26<br>Three groups based on academic performance and extracurricular activities.	Respondents were asked to discuss their own understandings of 'feeling well' and 'feeling good'.	Interpretive epistemology<br>Triangulation: group discussions, individual interviews, observational data.	Abductive strategy<br>Identification of broad topic areas<br>Thematic analysis<br>Theoretical analysis<br>Comparison of observations and accounts	Two key themes:<br>• Being happy<br>Knowing I can do something<br>Looking good<br>Talking to others<br>• Having fun<br>It's just having a laugh<br>Having fun at others<br>Having fun with friend

Reference	Country	Aim	Sample	SRH/health question(s)	Data collection method	Data analysis method	Key findings
			Professionals N=18				• Some gender differences

Supplemental file 6. Description of included studies by type of study question, data collection method, and study population

The 21 included studies varied across the following measures: type of study question, data collection methods, and study population. The relationship between these categories is shown in Figure 1.

1) Studies that investigated the conceptualisation of health in the survey context; study question includes the word "health".
In those studies, young people were asked to discuss the reasoning when they answered general health question in the survey (43, 44). Välimaa (44) used the SRH question as applied in the Finnish 1997 Health Behaviours in School-aged Children (HBSC) survey. Joffer et al. (43) contrasted the conceptualisation of health when the survey question included the word "health" or "feel". Both studies included respondents from the general population[3] and used interviews as a data collection method.

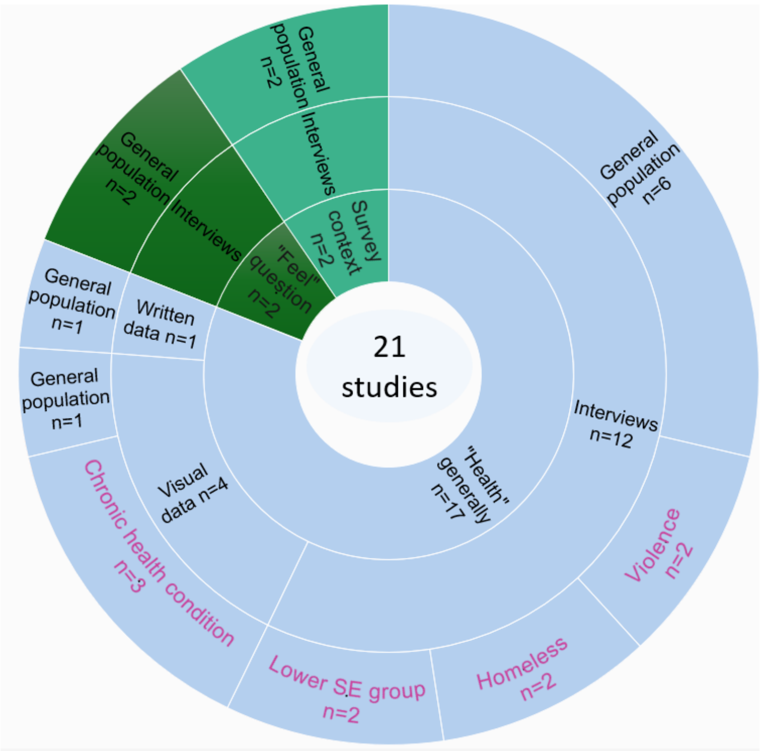

Figure 1. Included studies by the type of study question (inner circle), data collection method, and study population (outer circle)

2) Studies that investigated the concept of health in the survey context; study question includes the word "feel". The study by Joffer et al. (43) as described above, is included in this category.

3) Studies that investigated the concept of health generally; the study question includes the word "health".
17 studies were included in this category (45, 46, 47, 48, 49, 50, 51, 52, 53, 54, 55, 56, 57, 58, 59, 60, 61). The study by O'Higgins et al. (56) was partly motivated by the utilisation of self-reported health measure in health research; other studies were driven by the motivation to develop successful health interventions and policies (46, 47, 48, 53, 55, 57, 59, 61). The majority of studies used different types of interviews (45, 46, 49, 54, 56, 57, 58, 59, 60, 61), four studies (46, 55, 60, 61)

[3] In this study, "general population" refers to the sample recruited without defining specific characteristics of interest such as having a certain health condition, living in a deprived area, or belonging to certain ethnic group.

complemented interview data with visual methods, two studies also used observations (60, 61). Four studies used visual methods that were complemented by written or oral descriptions of the photographs and drawings (47, 50, 51, 52). The study by Hariharan et al. (53) is the only included study that used data from open-ended written questions. Three studies targeted adolescents with chronic health conditions and used the same study design (50, 51, 52). Two studies investigated perceptions of health among homeless young people (49, 54), and young people with experience of violence (45, 60). Two studies recruited respondents from lower socio-economic groups (48, 53).

4) Studies that investigated the concept of health generally; study question includes the word "feel".

Studies by Spencer (63) and Larsson (62) are included in this category, both studies recruited respondents from the general population and used interviews as data collection methods.

Supplemental file 7. Quality of included studies

A (No or few flaws), B (Some flaws), C (Significant flaws), and D (Untrustworthy)

QF question	Joffer et al 2016 (43)	Välimaa 2000 (44)	Berman 1999 (45)	Borraccino et al 2019 (46)	Cetin et al 2012 (47)	Dow et al 2022 (48)	Flick et al 2007 (49)	Hager 1997 (50)	Hanna et al 1993 (51)	Hanna et al 1995 (52)	O' Higgins et al 2010 (56)	Parvizy et al 2008 (58)	Randell et al 2016 (59)	Hariharan et al 2019 (53)	Karabanow et al 2007 (54)	Larsson et al 2012 (62)	Ott et al 2011 (57)	Woodgate et al 2010 (61)	Michaelson et al 2016 (55)	Spencer 2013 (63)	Sasakamoose et al 2016 (60)
**1**	A	A	A	A	A	B	A	A	A	A	A	B	A	A	C	A	B	A	A	A	B
**2**	A	A	B	A	B	B	B	A	A	A	B	B	A	A	C	B	B	A	A	A	C
**3**	A	A	A	A	B	A	B	A	A	A	A	A	A	A	B	B	B	A	A	A	A
**4**	A	A	B	B	C	B	D	A	B	A	B	B	A	B	A	A	C	C	A	A	C
**5**	A	A	A	A	B	A	C	A	B	A	B	B	A	A	A	A	B	B	A	A	B
**6**	A	A	A	B	C	B	C	A	C	A	B	C	B	C	B	C	C	C	B	A	C
**7**	B	B	B	A	C	C	C	A	B	A	C	B	B	B	A	A	C	B	A	A	C
**8**	A	A	B	B	B	B	B	A	B	B	B	B	A	C	A	A	B	B	A	A	C
**9**	A	A	A	A	A	A	C	A	A	A	C	B	B	B	C	B	B	B	A	A	D
**10**	A	A	A	B	C	C	A	A	C	B	A	C	B	B	B	C	C	C	A	B	C
**11**	A	A	A	B	B	B	B	A	A	B	A	C	B	A	B	B	C	B	C	A	B
**12**	A	A	A	B	B	A	A	A	A	B	B	C	A	A	B	A	B	A	A	A	B
**13**	A	A	B	B	B	B	A	A	B	B	B	B	A	A	B	A	B	A	A	A	B
**14**	A	A	A	A	A	B	A	A	A	A	B	B	A	A	B	A	A	A	A	A	B
**15**	B	B	B	C	C	A	C	A	B	B	B	B	A	B	C	A	B	B	A	A	C
**16**	A	B	C	A	D	C	D	A	B	A	C	C	A	C	C	A	B	C	B	B	B
**17**	A	A	C	B	C	B	C	A	B	A	B	C	B	C	C	C	C	B	B	A	D

Supplemental file 8. Stages of thematic synthesis

**Phase 1**<br>**Coding of included studies**<br>**n=124 codes**		**Phase 1-2**<br>**Refining codes**<br>**n=66**		**Phase 2**<br>**Descriptive themes**	**Phase 2-3**<br>**Refining descriptive themes into analytical themes**<br>**First draft of themes**	**Phase 3**<br>**Second draft of themes**	**Analytical themes and sub-themes**
1. Studies that investigated conceptualisation of health in the survey context; study question includes word "health"<br>2. Studies that investigated the concept of health in the survey context; study question includes the word "feel"	The content of codes compared and refined	13 codes<br>13 codes	Similar codes organised into descriptive themes	Four themes<br>Three sub-themes<br>Two themes<br>Six sub-themes	**'Health' and 'feel' in the surveys: domination of different dimensions of health**<br>*How healthy do you think you are: illnesses, symptoms and health behaviours*<br>*The 'feel' question: domination of mental dimension and holistic views*	**Health in context: physical, informational, and cultural environments**<br>*Environment, housing, and safety*<br>*Material resources*<br>*Informational context and technology*<br>*Traditions, culture and religion*<br>**Dimensions of health and their intersectionality**<br>*Physical health: Health behaviours, looking good, and functionality*<br>*Social dimension: relationships with friends, family, and (in) school*<br>*Mental dimension: emotions and subjectivity of health*<br>*Intersectionality of health*<br>**Striving for balance: challenges and coping strategies**<br>**"Health" and "Feel" in the surveys: domination of different dimensions of health**	**Dimensions of health**<br>*Physical dimension: health behaviours, functionality, and physical appearance*<br>➢ *Survey question: "How is your health in general?"*<br>*Social dimension: relationships with friends, family, and (in) school*<br>*Mental dimension: emotions and subjectivity of health*<br>*Intersectionality of health and striving for balance*<br>➢ *Survey question: "How do you feel?"*<br>**Health in context**<br>*Physical context: environment, housing, and safety*<br>*Material resources*<br>*Informational context and technology*<br>*Cultural environment: traditions, culture and religion*
3. Studies that investigated the concept of health generally; study question includes the word "health"<br>4. Studies that investigated the concept of health generally; study question includes the word "feel"		29 codes<br>11 codes		Seven themes<br>24 sub-themes<br>Four themes<br>Nine sub-themes	**Health is a complex (holistic) phenomenon interwoven in everyday life**<br>*Health in the context: physical, informational, and cultural environments*<br>• *Great outdoors, violence, and housing*<br>• *Material resources*<br>• *Informational context and technology*<br>• *Traditions*, culture and religion<br>*Different types of health: Physical, mental (emotional), and social dimension*<br>• *Health behaviours, looking good, and functioning*<br>• *Social dimension: relationships with friends, family, and (in) school*<br>• *Mental dimension: emotions and subjectivity of health*<br>• *Subjectivity of health: "You can be healthy without being completely healthy"*<br>*Finding balance: challenges and coping strategies*		

Supplemetal file 9. Contributions of included studies to analytical themes and sub-themes

Two analytical themes, eight sub-themes, and main factors	Berman (45)	Borraccino et al (46)	Cetin et al (47)	Dow et al (48)	Flick et al (49)	Hager (50)	Hanna et al (51)	Hanna et al (52)	Hariharan et al (53)	Joffer et al (43)	Karabanow et al (54)	Larsson et al (62)	Michaelson et al (55)	O´Higgins et al (56)	Ott et al (57)	Parvizy et al (58)	Randell et al (59)	Sasakamoose et al(60)	Spencer (63)	Välimaa (44)	Woodgate et al (61)
**DIMENSIONS OF HEALTH**																					
Physical dimension*: health behaviours, functionality, and physical appearance*																					
Diet																					
Physical activity and/or fitness																					
Medical aspects																					
Alcohol, tobacco, or substance use																					
Survey question: "How is your health in general?"																					
Social dimension: *relationships with friends, family, and (in) school*																					
Supportive relationships																					
Studies and school																					
Mental dimension*: emotions and subjectivity of health*																					
Happiness or fun																					
Stress and/or tiredness																					
Intersectionality of health *and striving for balance*																					
Survey question: "How do you feel?"																					
**HEALTH IN CONTEXT**																					
Physical context: environment, housing, and safety																					
Material resources																					
Informational context and technology																					
Cultural environment: traditions, culture and religion																					

Supplemental file 10. Original quotes in Välimaa 2000, translation from Finnish to English

Translated quotation	*Original quote in Finnish*
Girls focus group (44), page 126:<br>*" … that running up the stairs at school doesn't feel terrible. And that you are not very tired"*<br>*"If you have like a flu or you are hurting terribly"*<br>*"I thought more in a holistic way, that are you in a good condition."*<br>*"I didn't think like this at all. I just thought, if you are ill or if you have allergies or like that. I didn't think about the fitness side at all."*<br>*"I thought about all of this."*	*T: Mää ainakin semmoista, että jos koulussa portaita juoksee, että ei tunnu kauheelta. Ja sitt että ei oo kovin väsynyt.*<br>*No, että onks sulla jotain flunssaa tai oot sää hirveen kipee.*<br>*T: Mää ajattelin enemmänkin sillei kokonaisesti, että oot sää hyvässä kunnossa.*<br>*T: Mää en ajatellu taas sillei ollenkaan. Mää ajattelin vaan, että oot sää kipee tai onko sulla allergioita tai tällasta. En mää mitään kuntopuolta ajatelu.*<br>*T: Mää katoin ne sillei kaikki.*
Boys focus group (44):<br>*"So there is nothing permanent like compulsive movements or rheumatism …"*<br>*"I rather thought about general fitness. And of course, what if you have a skin cancer …" (page 127)*	*P: No ei oo mitään semmoista pysyvää jotain pakkoliikettä tai reumaa tai...*<br>*[1] P: Peruskuntoa pikemminkin siinä mietin. Ja sit sitä tietenkin, että mitä jos sairastaakin ihosyöpää …*
"In a way, if relationships are not okay, so yes it can influence general mood"<br>*"But I did not count this as health"*<br>*"Neither did I in this way"*<br>*"You don't think about this so often"*<br>*"You think like in a concrete way if you are healthy. Not like emotional aspects, although maybe should have thought. If you are terribly depressed and like this, it will influence health very much." (44) (page 128)*	*: Kyllähän sekin tavallaan, että jos ihmissuhteet ei oo kunnossa, niin kyllä se vähän*<br>*voi vaikuttaa yleiseen mielialaankin. T: Mut en mää sitä laskenu siihen terveyteen. T: En määkään sillä tavalla. T: Harvemminhan sitä miettii niinkun sitä. T: Sen aattelee jotenkin niinkun konkreettisesti sillei oot sä nyt terve. Ei siinä ajatellu mitään semmoisia tunnepuolia sun muita, vaikka ne nyt ehkä oiskin pitäny ajatella. Jos sää oot hirveen masentunu ja tällei niin kyl se terveyteenkin vaikuttaa hirveesti.*
"Many things influence health, mental balance, physical balance – everything. There are so many things, you cannot say what influences it and what doesn't." (44) (page 128)	*Terveyteen vaikuttaa moni asia, mielen tasapaino, ruumiin tasapaino - kaikki. Siis siinä on niin monta asiaa, ettei sitä nyt yhtäkkiä voi sanoo, et mikä siihen vaikuttaa ja mikä ei.*
Boy: "Many things influence health, mental balance, physical balance – everything. There are so many things, you cannot say what influences it and what doesn't."	*Terveyteen vaikuttaa moni asia, mielen tasapaino, ruumiin tasapaino - kaikki. Siis siinä on niin monta asiaa, ettei sitä nyt yhtäkkiä voi sanoo, et mikä siihen vaikuttaa ja mikä ei.*

REFERENCES SUPPLEMENTARY MATERIAL

1. Tong A, Flemming K, McInnes E, Oliver S, Craig J. Enhancing transparency in reporting the synthesis of qualitative research: ENTREQ. BMC Med Res Methodol. 2012;12(1):181.
2. Spencer L, Ritchie J, Lewis J, Dillon L, Research NCfS. Quality in Qualitative Evaluation: A framework for assessing research evidence 2003.
3. Aho AC, Hultsjo S, Hjelm K. Health perceptions of young adults living with recessive limb-girdle muscular dystrophy. J Adv Nurs. 2016;72(8):1915-25.
4. Barco Leme AC, Fisberg RM, Baranowski T, Nicklas T, Callender CS, Kasam A, et al. Perceptions About Health, Nutrition Knowledge, and MyPlate Food Categorization Among US Adolescents: A Qualitative Study. J Nutr Educ Behav. 2021;53(2):110-9.
5. Buck JS, Ryan-Wenger NA. Early Adolescents' Definition of Health: The Development of a New Taxonomy. Journal of Theory Construction & Testing. 2003;7(2):50-5.
6. Burrows L, Wright J, McCormack J. Dosing up on food and physical activity: New Zealand children's ideas about 'health'. The Health Education Journal. 2009;68(3):157-69.
7. Burrows L, Wright J, Jungersen-Smith J. “Measure Your Belly.” New Zealand Children’s Constructions of Health and Fitness. Journal of Teaching in Physical Education. 2002;22(1):39-48.
8. Caluzzi G, MacLean S, Livingston M, Pennay A. "No one associates alcohol with being in good health": Health and wellbeing as imperatives to manage alcohol use for young people. Sociol Health Illn. 2021;43(2):493-509.
9. Cronley C, Keaton C, Hopman DD, Nelson LP. "I run inside the buildings": adolescents' perceptions of physical health and nature in family homeless shelters. Journal of Children & Poverty. 2019;25(2):131-49.
10. De Moura SL, Harpham T, Lyons M. The social distribution of explanations of health and illness among adolescents in Sao Paulo, Brazil. J Adolesc. 2003;26(4):459-73.
11. Dixit A, Miner EM, Wiehe SE, McHenry MS. Adolescent Burmese refugees perspectives on determinants of health. Journal of Immigrant and Minority Health. 2018;20(2):370-9.
12. Friderichs M. " They do Think About Health" -Health, Culture and Identity in Katherine. PhD Thesis: Charles Darwin University (Australia); 2018.
13. Glozah FN. Exploring Ghanaian adolescents’ meaning of health and wellbeing: A psychosocial perspective. Int J Qual Stud Health Well-being. 2015;10.
14. Harris J, Cale L, Duncombe R, Musson H. Young people's knowledge and understanding of health, fitness and physical activity: issues, divides and dilemmas. Sport Education and Society. 2018;23(5):407-20.
15. Hinton RL, Earnest J. Beyond risk factors to lived experiences: young women's experiences of health in Papua New Guinea. Rural Remote Health. 2009;9(4):1257.
16. Hobin E, Anderson A. Middle-school students' concepts of health in Ontario and the British Virgin Islands and the implications for school health education. Physical & Health Education Journal. 2008;74(2).
17. Hsin M-C, Lin C-Y, Li H-Y, Lin S-Y. Students' conceptions of health: A cross educational stage survey. Heliyon. 2020;6(11):e05383.
18. Isaak CA, Marchessault G. Meaning of Health: The Perspectives of Aboriginal Adults and Youth in a Northern Manitoba First Nations Community. Can. 2008;32(2):114-22.
19. Isma GE, Rämgård M, Enskär K. Perceptions of health among school-aged children living in socially vulnerable areas in Sweden. Front. 2023;11.
20. Kroh J, Tuppat J, Gentile R, Reichelt H. How do Children Rate Their Health? An Investigation of Considered Health Dimensions, Health Factors, and Assessment Strategies. Child Indicators Research. 2023;16(6):2545-80.
21. Kefford CH, Trevena LJ, Willcock SM. Breaking away from the medical model: perceptions of health and health care in suburban Sydney youth. Med J Aust. 2005;183(8):418-21.
22. Martin S, Horgan D, Scanlon M, Eldin N, O'Donnell A. Including the voices of children and young people in health policy development: An Irish perspective. HEALTH EDUCATION JOURNAL. 2018;77(7):791-802.
23. McCloughen A, Foster K, Kerley D, Delgado C, Turnell A. Physical health and well-being: Experiences and perspectives of young adult mental health consumers. Int J Ment Health Nurs. 2016;25(4):299-307.
24. Millstein SG, Irwin CE. Concepts of health and illness: different constructs or variations on a theme? Health Psychology. 1987;6(6):515.
25. Natapoff JN. Children's views of health: a developmental study. Am J Public Health. 1978;68(10):995-1000.
26. Pang B, Alfrey L, Varea V. Young Chinese Australians' subjectivities of "health' and "(un)healthy bodies'. Sport Education and Society. 2016;21(7):1091-108.
27. Pfister G, With-Nielsen N, Lenneis V. Health discourses, slimness ideals, and attitudes to physical activities Perspectives of young women in Denmark. German Journal of Exercise and Sport Research 2017;47(1):15-24.
28. Piko BF, Bak J. Children's perceptions of health and illness: images and lay concepts in preadolescence. Health Education Research. 2006;21(5):643-53.
29. Quarmby T. Exploring the role of the family in the construction of young people's health discourses and dispositions. Educational Review. 2013;65(3):303-20.
30. Ravenell JE, Johnson WE, Whitaker EE. African-American men's perceptions of health: A focus group study. Journal of the National Medical Association. 2006;98(4):544-50.
31. Secor-Turner M, Randall BA, Mudzongo CC. Barriers and Facilitators of Adolescent Health in Rural Kenya. J Transcult Nurs. 2016;27(3):270-6.

32. Singletary JH, Bartle CL, Svirydzenka N, Suter-Giorgini NM, Cashmore AM, Dogra N. Young people's perceptions of mental and physical health in the context of general wellbeing. Health Education Journal. 2015;74(3):257-69.
33. Sundar TKB, Sargenius H, Kvarme LG, Sparboe-Nilsen B. Norwegian pre-service teacher students' and public health nursing students' views on health – a qualitative study of students' perceptions. Int J Qual Stud Health Well-being. 2024;19(1):2322705.
34. Zullig KJ, Valois RF, Drane JW. Adolescent distinctions between quality of life and self-rated health in quality of life research. Health Qual Life Outcomes. 2005;3:64.
35. van der Meer AS, Durlach F, Szota K, Christiansen H. "I can't describe how I could get better, but I would like to" - Conception of health and illness of refugee youth in Germany. Front Psychol. 2023;14:1107889.
36. Velardo S, Drummond M. Australian children's discourses of health, nutrition and fatness. Appetite. 2019;138:17-22.
37. Walker T, Molenaar A, Palermo C. A qualitative study exploring what it means to be healthy for young Indigenous Australians and the role of social media in influencing health behaviour. Health Promot J Aust. 2021;32(3):532-40.
38. Wang W. Mainland Chinese students' concept of health. Asia Pacific Journal of Public Health. 2004;16(2):89-94.
39. Woodgate RL, Skarlato O. It is about being outside': Canadian youth's perspectives of good health and the environment. Health Place. 2015;31:100-10.
40. Wright J, Burrows L. "Being Healthy": The discursive construction of health in New Zealand children's responses to the National Education Monitoring Project. Discourse: Studies in the Cultural Politics of Education. 2004;25(2):211-30.
41. Wright J, O'Flynn G, Macdonald D. Being Fit and Looking Healthy: Young Women's and Men's Constructions of Health and Fitness. Sex Roles. 2006;54(9):707-16.
42. Yu C, Lou C, Cheng Y, Cui Y, Lian Q, Wang Z, et al. Young internal migrants' major health issues and health seeking barriers in Shanghai, China: a qualitative study. BMC Public Health. 2019;19(1):336.
43. Joffer J, Jerden L, Ohman A, Flacking R. Exploring self-rated health among adolescents: a think-aloud study. BMC Public Health. 2016;16:156.
44. Välimaa R. Nuorten koettu terveys kyselyaineistojen ja ryhmähaastattelujen valossa: University of Jyväskylä; 2000.
45. Berman H. Health in the aftermath of violence: a critical narrative study of children of war and children of battered women. Can J Nurs Res. 1999;31(3):89-109.
46. Borraccino A, Pera R, Lemma P. "What being healthy means to me": A qualitative analysis uncovering the core categories of adolescents' perception of health. PLoS ONE. 2019;14(6):e0218727.
47. Cetin G, Özarslan M, Işık E, Eser H. Students' views about health concept by drawing and writing technique. 2012.
48. Dow M, Murrin C, O'Malley G, Brinkley A, Bel-Serrat S. A qualitative study exploring the perceptions of health among pre-teen girls from disadvantaged communities in Dublin. Children & Society. 2022.
49. Flick U, Röhnsch G. Idealization and Neglect: Health Concepts of Homeless Adolescents. Journal of Health Psychology. 2007;12(5):737-49.
50. Hager MS. Exploring the Concept of Health Among Rural Adolescents with Insulin Dependent Diabetes Mellitus Using Photography as a Facilitator of Communication [M.S.]. School of Nursing: University of Wyoming; 1997.
51. Hanna KM, Jacobs P. The Use of Photography to Explore the Meaning of Health among Adolescents with Cancer. Issues Compr Pediatr Nurs. 1993;16(3):155-64.
52. Hanna KM, Jacobs PM, Guthrie D. Exploring the Concept of Health Among Adolescents With Diabetes Using Photography. J Pediatr Nurs. 1995;10(5):321-7.
53. Hariharan M, Monteiro SR, Asha D, Rao CR. Perceptions of Health: a Developmental Trend in Indian School Children. Child Indicators Research. 2019;12(4):1351-68.
54. Karabanow J, Hopkins S, Kisely S, Parker J, Hughes J, Gahagan J, et al. Can You Be Healthy on the Street?: Exploring the Health Experience of Halifax Street Youth. Canadian Journal of Urban Research. 2007;16(1):12-32.
55. Michaelson V, Pickett W, Vandemeer E, Taylor B, Davison C. A mixed methods study of Canadian adolescents' perceptions of health. Int J Qual Stud Health Well-being. 2016;11(1):32891.
56. O'Higgins S, Sixsmith J, Gabhainn SN. Adolescents' perceptions of the words "health" and "happy.". Health Education. 2010;110(5):367-81.
57. Ott MA, Rosenberger JG, McBride KR, Woodcox SG. How do adolescents view health? Implications for state health policy. J Adolesc Health. 2011;48(4):398-403.
58. Parvizy S, Ahmadi F, Nikbakht Nasrabad A. An identity-based model for adolescent health in the Islamic Republic of Iran: a qualitative study. EMHJ-Eastern Mediterranean Health Journal, 14 (4), 869-879, 2008. 2008.
59. Randell E, Jerden L, Ohman A, Flacking R. What is Health and What is Important for its Achievement? A Qualitative Study on Adolescent Boys' Perceptions and Experiences of Health. Open Nurs J. 2016;10:26-35.
60. Sasakamoose J, Scerbe A, Wenaus I, Scandrett A. First Nation and Métis Youth Perspectives of Health. Qualitative Inquiry. 2016;22(8):636-50.
61. Woodgate RL, Leach J. Youth's Perspectives on the Determinants of Health. Qualitative health Research. 2010;20(9):1173-82.
62. Larsson M, Sundler AJ, Ekebergh M. Beyond self-rated health: The adolescent girl's lived experience of health in Sweden. The Journal of School Nursing. 2012;29(1):71-9.
63. Spencer G. Young people's perspectives on health: Empowerment, or risk? Health Education. 2013;113(2):115-31.
